# Cell-type-specific molecular characterization of cells from circulation and kidney in IgA nephropathy with nephrotic syndrome

**DOI:** 10.3389/fimmu.2023.1231937

**Published:** 2023-10-16

**Authors:** Qilin Chen, Huimin Jiang, Rong Ding, Jinjie Zhong, Longfei Li, Junli Wan, Xiaoqian Feng, Liping Peng, Xia Yang, Han Chen, Anshuo Wang, Jia Jiao, Qin Yang, Xuelan Chen, Xiaoqin Li, Lin Shi, Gaofu Zhang, Mo Wang, Haiping Yang, Qiu Li

**Affiliations:** ^1^ Department of Nephrology, Children’s Hospital of Chongqing Medical University, Chongqing, China; ^2^ National Clinical Research Center for Child Health and Disorders, Ministry of Education Key Laboratory of Child Development and Disorders, Chongqing, China; ^3^ Chongqing Key Laboratory of Pediatrics, Chongqing, China; ^4^ Nanjing Jiangbei New Area Biopharmaceutical Public Service Platform Co. Ltd, Nanjing, Jiangsu, China

**Keywords:** IgA nephropathy, nephrotic syndrome, single-cell RNA sequencing, peripheral blood mononuclear cells, podocyte

## Abstract

Nephrotic syndrome (NS) is a relatively rare and serious presentation of IgA nephropathy (IgAN) (NS-IgAN). Previous research has suggested that the pathogenesis of NS-IgAN may involve circulating immune imbalance and kidney injury; however, this has yet to be fully elucidated. To investigate the cellular and molecular status of NS-IgAN, we performed single-cell RNA sequencing (scRNA-seq) of peripheral blood mononuclear cells (PBMCs) and kidney cells from pediatric patients diagnosed with NS-IgAN by renal biopsy. Consistently, the proportion of intermediate monocytes (IMs) in NS-IgAN patients was higher than in healthy controls. Furthermore, flow cytometry confirmed that IMs were significantly increased in pediatric patients with NS. The characteristic expression of *VSIG4* and MHC class II molecules and an increase in oxidative phosphorylation may be important features of IMs in NS-IgAN. Notably, we found that the expression level of *CCR2* was significantly increased in the CMs, IMs, and NCMs of patients with NS-IgAN. This may be related to kidney injury. Regulatory T cells (Tregs) are classified into two subsets of cells: Treg1 (*CCR7* ^high^, *TCF7* ^high^, and *HLA-DR* ^low^) and Treg2 (*CCR7* ^low^, *TCF7* ^low^, and *HLA-DR* ^high^). We found that the levels of Treg2 cells expressed significant levels of *CCR4* and *GATA3*, which may be related to the recovery of kidney injury. The state of NS in patients was closely related to podocyte injury. The expression levels of *CCL2*, *PRSS23*, and genes related to epithelial-mesenchymal transition were significantly increased in podocytes from NS-IgAN patients. These represent key features of podocyte injury. Our analysis suggests that *PTGDS* is significantly downregulated following injury and may represent a new marker for podocytes. In this study, we systematically analyzed molecular events in the circulatory system and kidney tissue of pediatric patients with NS-IgAN, which provides new insights for targeted therapy in the future.

## Graphical Abstract

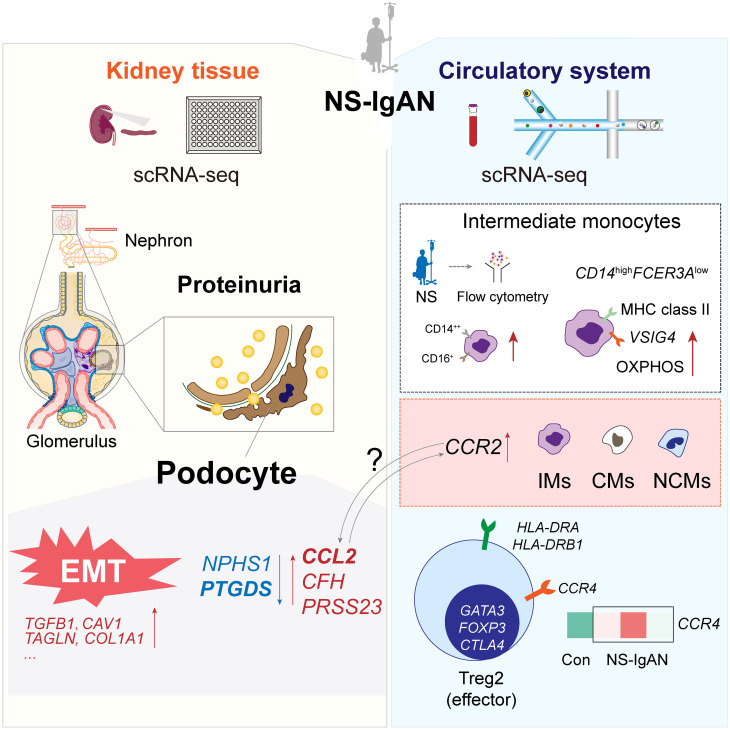

## 1 Introduction

IgA nephropathy (IgAN) is the most common form of primary glomerulonephritis worldwide (1). With the increased use of kidney biopsy in children, there is a growing concern related to pediatric patients with IgAN (2). The diagnostic hallmark of IgAN is the predominance of IgA deposits in the glomerular mesangium. The pathogenesis of IgAN remains unclear but it is considered an autoimmune disease. A “multi-hit’” hypothesis has been proposed to explain the pathogenesis of IgAN (1). This hypothesis outlines that increased levels of galactose-deficient IgA1 (Gd-IgA1), the production of autoantibodies, the deposition of immune complexes in the mesangial area of the glomeruli, and the secretion of cytokines, chemokines, and complements by mesangial cells ultimately lead to further kidney injury (3). In children, IgAN has long been considered to be a benign disease, with remission usually occurring after timely treatment (4). Nevertheless, patients with heavy proteinuria at biopsy often have a poor prognosis and there is a significant lack of therapeutic options for such patients (4). Nephrotic syndrome (NS) is a relatively rare and serious presentation of IgAN (NS-IgAN). This condition has a low incidence (4), ranging from approximately 4% to 10% (4, 5). Therefore, there is limited information relating to the molecular mechanism of NS-IgAN, particularly in pediatric populations.

NS-IgAN patients not only exhibit typical manifestations of nephrotic syndrome (e.g., edema, heavy proteinuria, and hypoproteinemia), they also show glomerular mesangial IgA deposition and extensive foot process effacement on kidney biopsy (6). When considering NS-IgAN, the presentation of the typical histological characteristics of IgAN can indicate the existence of two glomerular diseases: IgAN and “podocytopathy” (6). The injury and loss of podocytes are key factors that contribute to progressive proteinuria and filtration dysfunction in IgAN (7). In addition, mesangial-derived humoral factors, such as tumor necrosis factor, complement components, and angiotensin II, may alter glomerular permeability in the presence of proteinuria (8). Existing literature suggests that abnormal crosstalk between circulating immune cells and kidney cells may contribute to the occurrence and progression of IgAN. Systematic dissection of the molecular characteristics of the circulating immune system and kidney tissue will help us to identify the mechanisms underlying proteinuria and provide potential strategies for improving the treatment and prognosis of pediatric patients with NS-IgAN.

Single-cell transcriptome sequencing (scRNA-seq) technology can achieve unbiased and high-throughput transcriptome sequencing at the single-cell level and has been applied in clinical research for various diseases, including kidney and autoimmune diseases (9). Previous scRNA-seq studies of IgAN have attempted to identify the molecular features of kidney cells or circulating immune cells in IgAN (10–12). However, there is still a significant lack of research on the systematic identification of the changes in circulating immune cells and kidney cells that occur in IgAN (9). To provide valuable insight into the molecular features of local tissues and the circulatory system, we simultaneously performed scRNA-seq on kidney biopsies and peripheral blood mononuclear cells (PBMCs) from pediatric patients with NS-IgAN. We found that the composition of monocyte subsets was altered in NS-IgAN and this was accompanied by the increased expression of *CCR2*. In addition, we found that *PTGDS* was significantly downregulated in podocyte injury. Collectively, our research provides a new understanding of the molecular characteristics of cell types in NS-IgAN and may facilitate the development of new targeted therapies.

## 2 Materials and methods

### 2.1 Ethical approval

This study for scRNA-seq analysis was reviewed and approved by the Institutional Review Board of the Children’s Hospital of Chongqing Medical University (File Number: 2022 Research 124). The research for flow cytometric analysis was reviewed and approved by the Institutional Review Board of the Children’s Hospital of Chongqing Medical University. The File Number is 2022 Research 35. Written informed consent was obtained from all participants and their guardians.

### 2.2 Sample collection and patient details

In this study, we collected PBMCs from the blood and kidney tissues from three pediatric patients diagnosed with IgAN by renal biopsy, all of whom had IgAN with nephrotic-level proteinuria (NS-IgAN). The PBMCs were harvested from samples that remained after routine tests had been performed. Patients were classified as NGC, SGC, or LGC according to their glucocorticoid treatment at the time of sampling. We also included two kidney tissues as control kidney samples: one sample was Wilms’ tumor paracancerous tissue from a child (CTRL-1) while the other sample was from a resected duplex kidney (CTRL-2). All participants were enrolled from the Children’s Hospital of Chongqing Medical University, and each sample was collected for scRNA-seq individually. For comparative analysis, we downloaded scRNA-seq datasets from the Gene Expression Omnibus database. The kidney tissue datasets included one healthy adult (CTRL-3), four adult patients with IgAN (merged as HU-IgAN) from GSE171314, and PBMC datasets from three healthy children (Con-1, Con-2, Con-3) from GSE206295. Detailed information relating to the participants is shown in **Supplementary Table 1**. To further investigate the changes in monocyte subsets in NS patients, we recruited a cohort of 13 healthy children, 26 NS patients with proteinuria, and 6 NS patients who were in remission after treatment. We collected blood from samples that remained after routine testing for flow cytometry.

### 2.3 Preparation of single-cell suspensions

To prepare PBMCs, we collected 2 mL of venous blood in EDTA collection vessels, which were then taken to the laboratory on ice. PBMCs were isolated using Ficoll medium (TBD, Tianjin, China) and cryopreserved according to the 10X genomics recommended protocol (CG00039). To dissociate the kidney tissue into single-cell suspensions, each fresh kidney sample was washed three times with Hanks’ balanced salt solution and immediately stored in GEXSCOPE tissue preservation solution (Singleron Biotechnologies, Nanjing, China) at 2–8°C. Then, the tissue was cut into small pieces and digested in 1 mL of Tissue Dissociation Mix (Singleron Biotechnologies) at 37°C for 15 minutes before being passed through a 40 μm filter. After centrifugation at 3500 g for 5 minutes, cell pellets were resuspended in 1 mL of cold PBS. To remove red blood cells, 2 mL of GEXSCOPE Red Blood Cell Lysis Buffer (Singleron) was added into the cell suspension and incubated at 25°C for 10 minutes. Cells were then centrifuged at 300 g for 5 min and resuspended in cold PBS. Next, cells were stained with trypan blue (Beyotime, Shanghai, China) and counted with a TC20 automated cell counter (Bio-Rad, California, USA). Sample processing and analysis were permitted once cell viability exceeded 85%.

### 2.4 scRNA-seq library construction and sequencing

Several different sequencing methods were used for the samples of PBMCs and kidneys. For PBMCs, each sample was diluted to a final concentration of 700–1200 cells µl^−1^ and loaded onto a Chromium Single Cell Controller (10X Genomics, San Francisco, USA). The libraries for scRNA-seq were constructed using a Chromium Next GEM Single Cell 3´ GEM, Library and Gel Bead Kit v3.1 (10X Genomics) and then sequenced using an Illumina NovaSeq 6000 system. For each kidney sample, the single-cell suspension was adjusted to a concentration of approximately 300 cells µl^−1^. A GEXSCOPE Single Cell RNA Library Kit (Singleron Biotechnologies) was then used to construct a single-cell RNA-seq library for kidney samples. The libraries were then sequenced with an Illumina HiSeq X 10 system. Each sample of PBMCs and kidney tissue was processed independently.

### 2.5 scRNA-seq data processing

Raw sequencing reads from PBMCs were processed using Cell Ranger (version 6.0.0), including demultiplexing, genome alignment (GRCh38), barcode counting, and unique molecular identifier (UMI) processing. Similarly, raw data from the kidney tissues were processed by Celescope (version 1.10.0). We then used the Seurat (version 4.1.0) package to perform downstream analysis. To exclude low-quality cells, the cells were filtered by gene counts and UMI counts; cells with a high mitochondrial content were removed. Detailed information relating to the quality control (QC) threshold settings is given in **Supplementary Table 2**. After cell filtering, 53,571 PBMCs and 47,602 renal cells were captured for downstream analysis. Next, we used DoubletFinder (version 2.0.3) to identify doublets and removed clusters with a high proportion of doublets. To remove batch-effects, we integrated sample datasets via the “integrate” function in Seurat. Principal component analysis (PCA) was then performed on the top 2000 highly variable genes; the top 20 PCs were used for subsequent analysis. We then used a graph-based clustering algorithm to identify clusters, thus allowing us to construct a K-Nearest Neighbor (KNN) graph by Euclidean distance. The Louvain algorithm was used to group cells and optimize modules. To display the distribution of cells by status, we used the uniform manifold approximation and projection (UMAP) algorithms to visualize clustered cells. First, cells were identified by SingleR (v1.6.1). This software compared the transcriptome of each cell cluster to various reference datasets (e.g., human primary cell atlas, Blueprint/ENCODE, Database of Immune Cell Expression, Novershtern hematopoietic data, and Monaco immune data). In order to exclude the influence of automatic assignment, we also manually adjusted the identities of clusters by combining data with the expression levels of canonical marker genes and existing annotated scRNA-Seq data (**Supplementary Tables 3–7**, **9**).

### 2.6 Differentially expressed genes and enrichment analysis

Differentially expressed genes (DEGs) were computed using the FindMarkers function of Seurat. DEGs were defined as genes detected in at least 10% of cells, with a threshold of 0.25 log fold change using the Two-tailed Wilcoxon Rank-Sum Test with *P* < 0.01 following Bonferroni correction. Gene Ontology (GO) and Kyoto Encyclopedia of Genes and Genomes (KEGG) enrichment analyses were performed with the Metascape web tool (www.metascape.org) and ClusterProfiler (version 4.2.2).

### 2.7 Calculation and Analysis of epithelial-mesenchymal transition scores

We used cell scores to evaluate the degree to which individual cells expressed a certain predefined expression gene set. This allowed us to define meaningful functions and states. The cell scores were calculated using the Seurat function “AddModuleScore”, which calculated the average expression of genes from the predefined gene set in the respective cell. The control gene sets were randomly selected based on aggregate expression level bins. The final gene set score was obtained by subtracting the control score from the predefined gene set score. We then used several well-defined EMT markers (**Supplementary Table 8**) to define the EMT score.

To assess the statistical significance of scores, for each NS-IgAN patient or HU-IgAN group, the EMT scores were compared with that of the control group (CTRL) using the two-tailed Mann-Whitney *U*-test. Differences with a *P* value <0.05 were considered significant, *P<0.05, **P<0.01, ***P<0.001, and ****P<0.0001.

### 2.8 Ligand−receptor analysis

CellphoneDB was used with default parameters to reveal changes in interactions between different cell types.

### 2.9 Pseudo-time analysis

R package Monocle2 (version 2.18.0) was used to perform pseudo-time analysis. To clarify the role of genes in cell fate decisions, branched expression analysis modeling (BEAM) from Monocle2 was applied.

### 2.10 GWAS expression analysis

The defined IgAN susceptibility genes were obtained based on previous genome-wide association studies (GWASs) in IgAN combined with NephQTL and eQTL (cf) online analysis tools, and were provided in **Supplementary Table 10**. Cell-type specific expression of IgAN susceptibility genes was calculated by the average relative logarithmic expression values.

### 2.11 Cell culture and treatment

Human immortalized podocytes were provided from the lab of Zhihong Liu, and the cells were cultured as previously described (13). Cells were grown at the permissive temperature of 33°C (in 5% CO_2_) and differentiated at 37°C (in 5% CO_2_). After differentiating for 7-14 days, podocytes were treated with 0.25 µg mL^-1^ doxorubicin (Sigma-Aldrich, Saint Louis, USA) and 0.5 µg mL^-1^ doxorubicin for 24 hours.

### 2.12 Immunofluorescence

The kidney tissue was dewaxed and then heat-mediated antigen repair was performed in sodium citrate solution (pH=6.0) for 15min, and blocked with goat serum. Cultured podocytes growing on a glass slide were fixed in 4% paraformaldehyde for 15 min. The sections and cells were incubated with the following primary antibodies: Prostaglandin D Synthase (PGDS) (ABclonal, Wuhan, China), anti-nephrin (PROGEN, Darmstadt, Germany) at 4°C overnight. Then incubated with the appropriate secondary antibody for 45 or 60 min at room temperature: anti-guinea pig IgG antibody conjugated with Alexa Fluor 568 (Invitrogen, California, USA), anti-rabbit IgG antibody conjugated with Alexa Fluor 633 (Invitrogen). Nuclei were counterstained with Hoechst (Thermo Fisher, Boston, USA). Fluorescence signals were viewed under a fluorescence microscope (Nikon A1R, Tokyo, Japan). NIS-Element (version 5.5) was used to quantify PGDS and nephrin staining intensity.

### 2.13 Flow cytometric analysis

200μL whole blood was incubated with CD14-PE (BioLegend, California, USA) and CD16-APC (BioLegend, California, USA) antibodies. After staining for 20 minutes at room temperature in the dark, erythrocytes (BD Pharmingen, New Jersey, USA) in the samples were lysed by incubation with lysing solution for 5 minutes. Following centrifugation (300g/5 minutes, 4°C) and washing with PBS, cells were then examined using BD FACSCanto™. The datasets were analyzed using FlowJo (version 10.4.2).

### 2.14 Statistical analysis

Data are expressed as means ± standard deviation (SD). The EMT scores and gene expression levels were analyzed by the two-tailed Mann-Whitney *U*-test in SPSS 26.0. Differences in staining intensity between the two groups were analyzed by the two-tailed Student’s *t* test. All other analyses involved the two-tailed Wilcoxon Rank-Sum Test. Significance was defined as *P<0.05, **P<0.01, ***P<0.001. No specific indication is given if data were not significant. Graphs were generated by ggplot2 (version 3.3.5) and pheatmap (version 1.0.12) in R.

## 3 Results

### 3.1 Construction of a cell atlas of renal tissue and circulating immune cells in children with NS-IgAN

**Figure 1:**
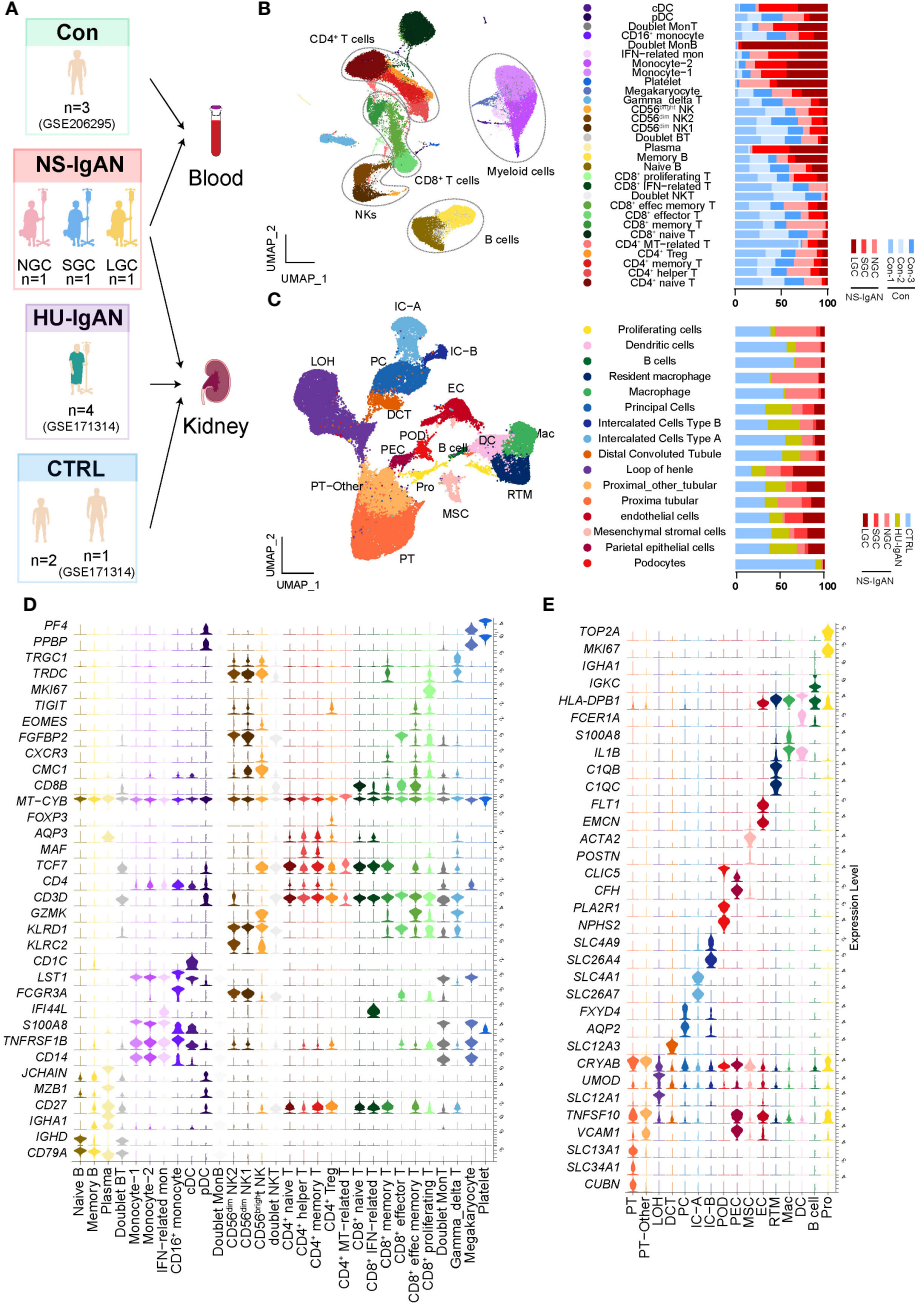
The landscape of PBMCs and kidney cells in NS-IgAN patients and healthy controls identified by single-cell transcriptomic analysis. **(A)** Schematic diagram of the study design for scRNA-seq. Con, n=3; NS-IgAN, n=3 (NGC, n=1; SGC, n=1; LGC, n=1); CTRL, n=3; HU-IgAN, n=4. **(B)** Distribution of 30 cell clusters in PBMCs. The figure on the left is a two-dimensional UMAP visualization of PBMCs. Different colors represent 30 clusters. The figure on the right is the percentage of each sample in each cell type. **(C)** Distribution of 16 cell types in kidney cells. The figure on the left is a two-dimensional UMAP visualization of kidney cells. Different colors represent 16 cell types. The figure on the right is the percentage of each sample in each cell type **(D)** Violin plot showing marker genes for each PBMC cell type. **(E)** Violin plot showing marker genes for each kidney cell type.

scRNA-seq was performed on PBMCs and kidney cells from three pediatric patients, all of whom had IgAN with nephrotic-level proteinuria, hypoalbuminemia, and hyperlipidemia (NS-IgAN) (**Figure 1A**; **Supplementary Table 1**). Kidney biopsy revealed the mesangial deposition of IgA and extensive or partial foot process effacement (**Supplementary Figures 1A–C**). It is worth noting that all three patients showed the pathological characteristics of mesangial hypercellularity. These patients had accepted different treatments at the time point of sampling, one patient was not on glucocorticoid (GC) therapy (NGC), one patient was on short-term GC therapy (SGC), and one patient was on long-term GC therapy (LGC) (**Supplementary Figures 1D–F**). Following the scRNA-seq of PBMCs, the raw data of three NS-IgAN patients and three healthy controls (Cons) from GSE206295 were merged and 53,571 PBMCs were captured following QC (**Figure 1B**; **Supplementary Table 2**). We annotated 30 cell types of PBMCs based on the expression of canonical markers, including CD4^+^ T cells (*CD3D*, *CD4*), CD8^+^ T cells (*CD3D*, *CD8A*), natural killer (NK) cells (*KLRB1*, *GNLY*), B cells (*CD19*, *CD79A*), myeloid cells (*CD68*, *LST1*), γδT cells (*CD3D*, *TRDC*), and megakaryocytes (*PPBP*, *PF4*) (**Supplementary Figure 2A**; **Supplementary Table 3**). For the kidney dataset, we downloaded data from GSE171314 as the HU-IgAN group, including four adult IgAN patients with hematuria and no nephrotic-level proteinuria (**Supplementary Table 1**). In addition, we performed scRNA-seq on the kidney tissues from two pediatric patients and included a single-cell public database (GSE171314) from one adult kidney as a control group (CTRL) (**Figure 1A**). The data from a total of ten kidney samples from three groups (CTRL, NS-IgAN, HU-IgAN) were integrated and 47,602 kidney cells were captured after QC (**Supplementary Table 2**). There were 26 clusters after dimension reduction (**Supplementary Figure 2B**). Then, 16 major cell types were annotated with the expression of canonical marker genes (**Figure 1C**; **Supplementary Table 4**). The expression levels of canonical marker genes for all cell types in the PBMCs and kidney cells are shown in **Figures 1D, E**.

### 3.2 The proportion of intermediate monocytes expressing MHC class II molecules is significantly increased in the disease

**Figure 2:**
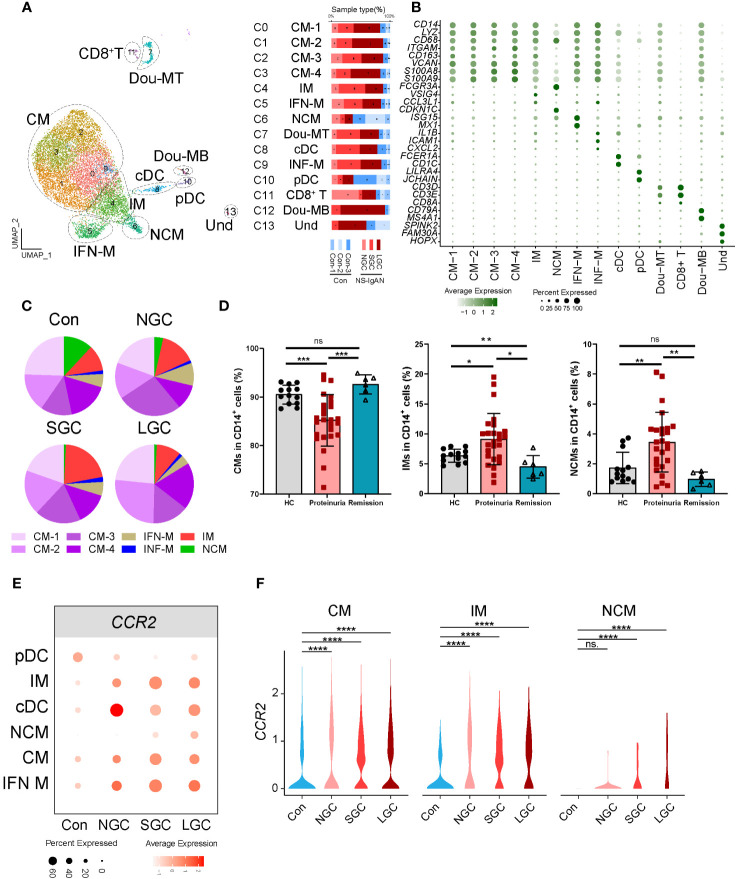
Molecular characterization of myeloid cells in NS-IgAN. **(A)** 13 clusters were visualized by UMAP plotting through re-clustering analysis of myeloid cells. **(B)** Violin plots showing expression of marker genes in 13 clusters. **(C)** The pie chart shows the proportion of different monocyte subsets in monocytes. **(D)** The proportion of monocyte subsets in NS patients, from left to right are CMs, IMs, and NCMs in flow cytometry. **(E, F)** Expression level of *CCR2* in monocyte subsets from different samples. All differences with *P* < 0.05 are indicated, *P<0.05, **P<0.01, ***P<0.001, and ****P<0.0001, ns means no significant difference.

Our investigation of the composition ratio of each sample in the same cell type found that the composition of SGC and LGC samples in myeloid cell types was prominent (**Figure 1B**). In response, we re-clustered the myeloid cell types into 14 cell clusters (**Figure 2A**). According to the expression of canonical marker genes for each cluster, we defined the cell types of clusters (**Figure 2B**, **Supplementary Table 5**). According to the expression levels of *CD14* and *FCER3A* (encoding CD16 protein), we respectively defined classical (CMs, *CD14* ^high^, *FCER3A* ^neg^), intermediate (IMs, *CD14* ^high^, *FCER3A* ^low^), and non-classical monocytes (NCMs, *CD14* ^low^, *FCER3A* ^high^) (**Figure 2B**) (14). Of these, the CMs were composed of four cell clusters. Cluster 5 expressed interferon-related genes (*ISG15*, *MX1*) and was defined as CD14^+^ IFN-related monocytes (IFN-Mon) (**Figure 2B**). Notably, we found that cluster 9 expressed *CCL4*, *IL1B*, *ICAM1*, and *CXCL2*. This cluster was defined as inflammatory monocytes (INF-M) (**Figure 2B**). Although the proportion of INF-M in myeloid cells did not differ significantly between disease and healthy samples (**Figure 2C**), we found that this group of cells was dominant in the disease group (**Figure 2A**).

**Figure 2C** shows the composition ratio of each monocyte subset to CD14^+^ cells in the controls and three cases. In NS-IgAN, the proportions of CM 1-4 and IMs tended to increase, and the proportion of NCMs tended to decrease; however, these trends were not statistically significant (**Figure 2C**, **Supplementary Figure 3A**). Previous evidence proved that monocyte subsets undergo changes in IgAN (15); our data suggested that similar changes may also exist in NS-IgAN. Since NS is the main clinical manifestation of NS-IgAN, we collected the peripheral blood of 26 NS patients with proteinuria, 6 NS patients with remission after treatment, and 13 healthy children to investigate changes in monocyte subsets in NS by flow cytometry. Monocytes were divided into classical monocytes (CMs, CD14^++^CD16^-^), intermediate monocytes (IMs, CD14^++^CD16^+^), and non-classical monocytes (NCMs, CD14^+^CD16^++^) through flow cytometry by the expression levels of CD14 and CD16 (**Supplementary Figure 3B**). The proportion of CMs in NS patients with proteinuria was significantly lower than that in healthy children, and the proportion of IMs and NCMs was significantly increased (**Figure 2D**). Following the glucocorticoid-induced remission of proteinuria, the proportion of CMs and NCMs returned to normal levels; the proportion of IMs was even lower than healthy controls (**Figure 2D**). Although the results of scRNA-seq and flow cytometry were different when compared between CMs and NCMs, the results consistently indicated a trend for an increased proportion of IMs. We found that IMs were characterized by high expression levels of *VSIG4*, *HLA-DPA1*, *HLA-DPB1*, and other MHC class II molecules (**Supplementary Figure 3C**). KEGG and GO enrichment analysis of the IMs showed that their function was mainly related to cytokine-cytokine receptor interaction, the chemokine signaling pathway, antigen processing and presentation, and MHC class II protein complex assembly (**Supplementary Figures 4A, B**). DEG analysis of IMs in the Cons and NS-IgAN groups showed that the expression of *VSIG4*, *LYZ*, *HLA-C*, *FN1*, and *FCGRT* was significantly increased in NS-IgAN (**Supplementary Figure 4C**). The significant up-regulation of DEGs in the IMs of NS-IgAN patients were related to oxidative phosphorylation by KEGG enrichment analysis (**Supplementary Figure 4D**). These results suggest that increased oxidative phosphorylation may be an important feature of IMs in NS-IgAN.

### 3.3 *CCR2* was significantly expressed in the IMs of NS-IgAN patients

DEG analysis of monocytes in the Cons and NS-IgAN groups showed that the expression levels of *CCR2* were significantly higher in the CMs (CM1-4), IMs, and NCMs of NS-IgAN patients (**Figures 2E, F**). CCR2 mediates monocyte chemoattractant recruitment to inflammatory regions and promotes the production of inflammatory cytokines (16). It has been reported that *Ccr2*-deficient mice with adriamycin-induced nephropathy showed reduced levels of injury, along with reduced macrophage and fibrocyte infiltration and inflammation in the kidney (17). To investigate the effect of high expression levels of *CCR2* on monocytes in NS-IgAN, we divided monocytes into *CCR2* ^+^ monocytes and *CCR2* ^-^ monocytes for further analysis. We found that the proportion of *CCR2* ^+^ monocytes was slightly increased in NS-IgAN, although this was not statistically significant (**Supplementary Figure 4E**). We also found that compared with *CCR2* ^-^ monocytes, *CCR2* ^+^ monocytes expressed high levels of *LYZ* and *HLA-DQA2* (**Supplementary Figure 4F**). *HLA-DQA2* encodes HLA class II alpha-chain proteins to constitute MHC class II molecules. A recent GWAS study of a Chinese IgAN cohort revealed significant associations between three HLA polymorphisms, thus indicating the extensive involvement of HLA-mediated immunity in IgAN development (18). Collectively these results suggested that the proportion of *CCR2* ^+^ monocytes is increased in NS-IgAN and that this may participate in the pathogenetic process.

### 3.4 GATA3^+^ effector Tregs expressed high levels of *CCR4* in NS-IgAN

**Figure 3:**
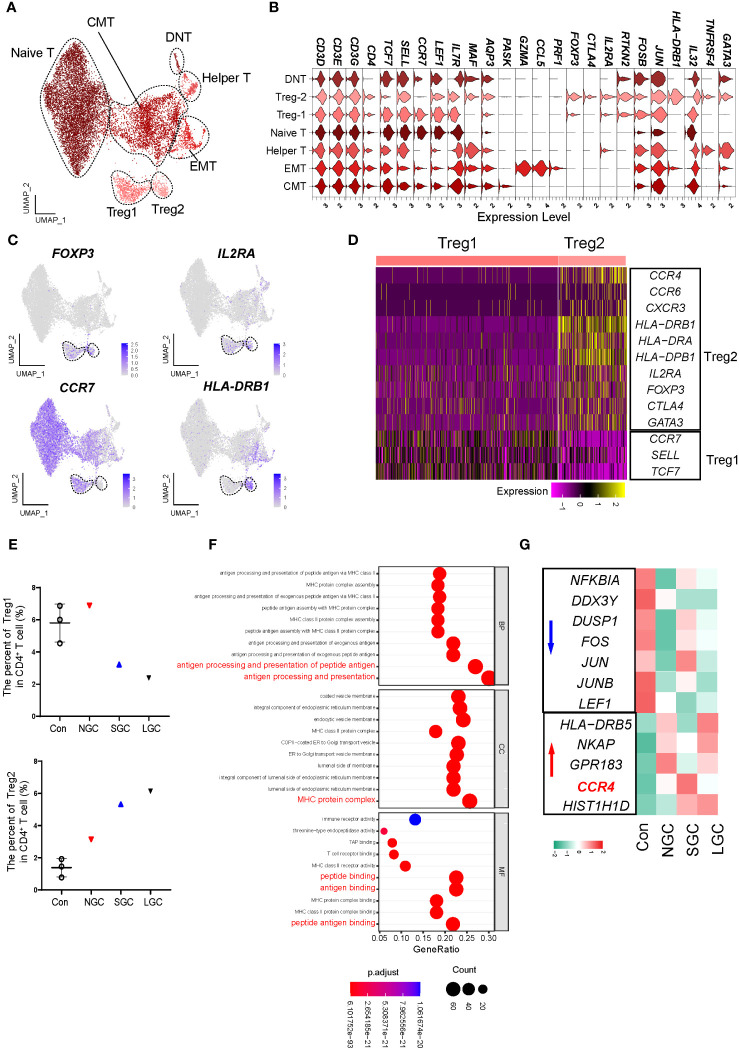
Molecular characterization of CD4^+^ T cells in NS-IgAN. **(A)** 7 distinct subsets were visualized by UMAP plotting through re-clustering analysis of CD4^+^ T cells. **(B)** Violin plots showing expression of marker genes in 7 subsets. **(C)** UMAP plots showing color-coded expression of four representative markers, *FOXP3*, *IL2RA*, *CCR7*, and *HLA-DRB1* in CD4^+^ T cells. **(D)** Heatmap exhibiting the differential expressing genes of Treg1 and Treg2. **(E)** The percent of Treg1 and Treg2 in CD4^+^ T cells. **(F)** GO terms identified by differential pathway enrichment and GO analysis via comparison of Treg2 cells vs. others. GO terms are labeled with name, and sorted by −log_10_(P) value. A darker color indicates a smaller P value. Interesting terms are labeled in red. **(G)** Heatmap exhibiting DEGs of Treg2 in Con, NGC, SGC, and LGC.

The proportion of Tregs in the peripheral blood of IgAN was significantly lower than that in controls, thus an increase in the proportion of Treg cells could improve clinical prognosis (19). We performed a re-clustering analysis of CD4^+^ T cells and identified 13 clusters (**Supplementary Figure 5A**); these were defined as eight cell types by the expression of canonical marker genes (**Figures 3A, B**; **Supplementary Table 6**). Of these, two clusters expressed *FOXP3* and *IL2RA*; we defined these as Treg1 and Treg2 cells (**Figure 3C**). According to the canonical marker genes expressed by different subsets of Tregs (20, 21), we found that Treg1 cells expressed high levels of *CCR7*, *SELL*, and *TCF7*; which may indicate that the cells are in a naive state; Treg2 cells expressed high levels of *CCR4* and HLA class II molecules, which may indicate that the cells are in an effector state (**Figure 3D**). The two groups of Treg cells consistently expressed some classical genes, including *TIGIT*, *IKZF2H*, and *RTKN2*, but also had molecular expression characteristics (**Supplementary Figures 5B, C**). We found that the proportion of Treg2 cells in NS-IgAN tended to be higher than that in the Cons group, while the proportion of Treg1 cells tended to be lower, although these differences were not statistically significant (**Figure 3E**). The results of GO enrichment analysis suggested that the functions of Treg2 cells were mainly related to peptide antigen binding, MHC protein complex, and antigen processing and presentation (**Figure 3F**). In Treg2 cells from NS-IgAN patients, we found that the expression levels of genes such as *FOS*, *JUN*, and *JUNB* were decreased, while those of *CCR4* were upregulated (**Figure 3G**). Recent research reported that a significant increase in the number of GATA3^+^ Tregs in the kidney was closely related to disease remission (22). CCR4 is known as an important chemokine receptor that promotes the infiltration of GATA3^+^ Tregs in the kidney during the later phases of injury (22). Our results suggest that there may be an increased proportion of CCR4^+^ GATA3^+^ Tregs in the circulation of NS-IgAN patients, thus indicating that dynamic changes of this special subset of Tregs may be involved in recovering from kidney injury.

Considering the important role of B cells in IgAN, we also performed a re-clustering analysis of B cells (**Supplementary Figure 6A**). We defined 12 clusters as 6 cell types by the expression of marker genes (**Supplementary Figures 6B, C**, **Supplementary Table 7**). Consistent with expectations, the *IGHA1* encoding IgA was mainly expressed in plasma cells. Pseudo-time analysis of B cells suggested that plasma cells at the end of differentiation were increasingly dominant in the disease, and expressed *IGHA1* and *IGHA2* (**Supplementary Figures 6D–G**). We compared the expression of key genes related to IgAN in diseased and healthy B cells; analysis suggested that *C1GALT1* expression was downregulated in IgAN; this may be related to the formation of Gd-IgA1 (**Supplementary Figure 6H**). In addition, the expression of IgG and IgA-related genes in the B cells of SGC and LGC patients was upregulated (**Supplementary Figure 6H**). Findings related to Treg cells and plasma cells are restrictive and limited by the number of captured cells, meaning that further research needs to be undertaken to validate these findings.

### 3.5 Podocytes in NS-IgAN expressed high levels of *CCL2* and EMT characteristics

**Figure 4:**
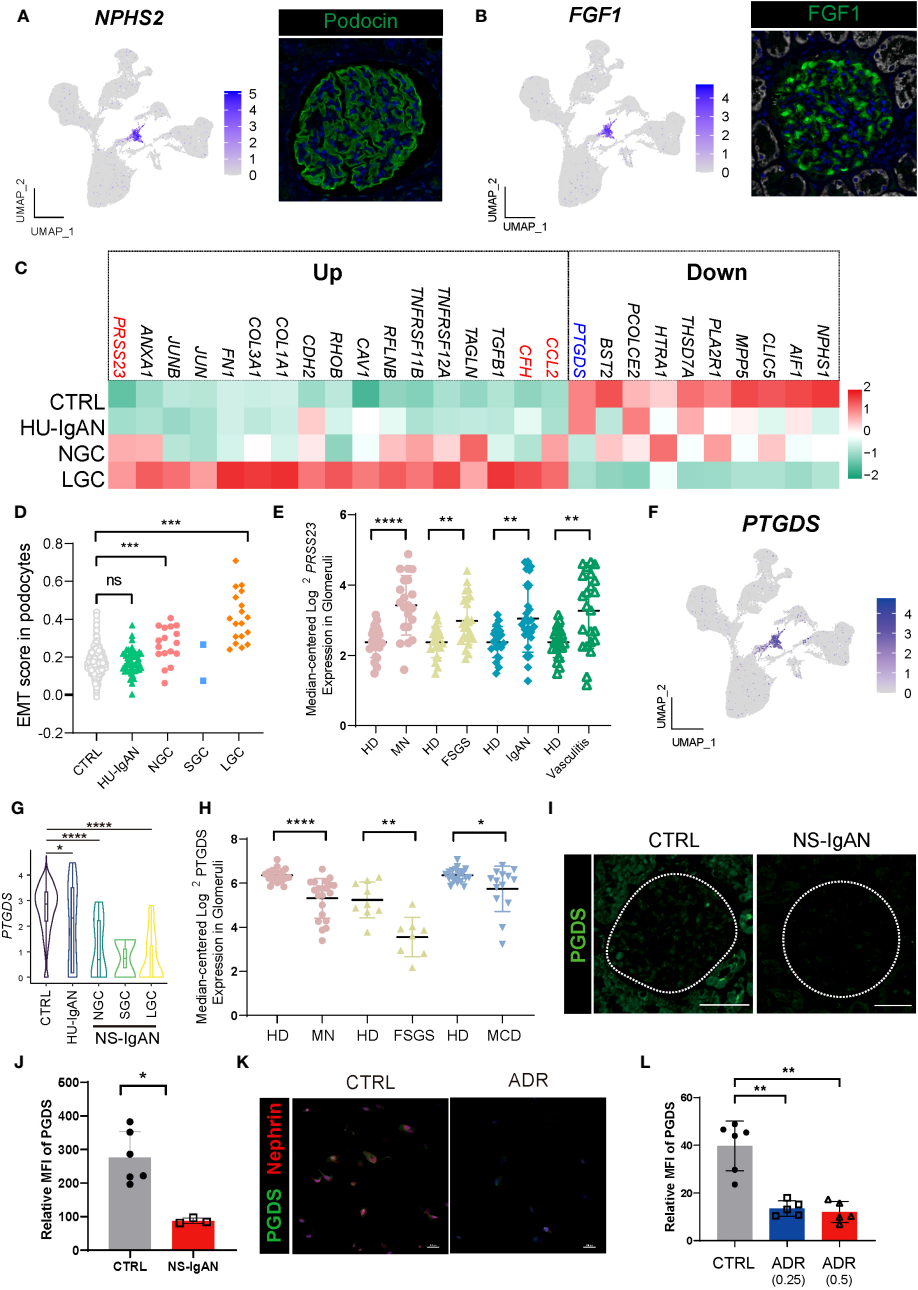
Molecular characterization of podocytes in NS-IgAN. **(A, B)** UMAP plots showing expression of *NPHS2* **(A)** and *FGF1* **(B)** in kidney and fluorescence staining of podocin and FGF1 in healthy kidney from The Human Protein Atlas database (https://www.proteinatlas.org/). **(C)** Heatmap showing up- and down-regulated DEGs of podocytes in the CTRL, HU-IgAN, NGC, and LGC groups. **(D)** EMT gene set scores for podocytes in CTRL, HU-IgAN, NGC, SGC, and LGC groups. **(E)** *PRSS23* mRNA expression in glomeruli of human biopsy specimens with pathological diagnosis of MN, FSGS, IgAN, or vasculitis disease compared with normal kidneys. Data are from previously published microarray studies by Ju et al. (2013) (23) and were subjected to further analysis using Nephroseq. A two-tailed Mann–Whitney *U* test was used for each comparison. All differences with *P* < 0.05 are indicated, *P<0.05, **P<0.01, ***P<0.001, and ****P<0.0001. **(F)** UMAP plots showing the expression of *PTGDS* in the kidney. **(G)** Statistical analysis of the expression level of *PTGDS* in podocytes in HU-IgAN, NGC, and LGC compared with CTRL. Since only 2 podocytes were captured in SGC patients, the comparison between SGC and CTRL was not performed. **(H)** *PTGDS* mRNA expression in glomeruli of human biopsy with pathological diagnosis of MN, FSGS, IgAN, or vasculitis disease compared with normal kidneys. Data are from previously published microarray studies by Ju et al. (2013) (23) and were subjected to further analysis using Nephroseq. **(I)** Immunofluorescence staining of PGDS in glomeruli of the NS-IgAN patient and the healthy control child. **(J)** Statistical analysis of PGDS glomerular mean fluorescence intensity quantified using NIS-Elements software. **(K)** Representative pictures of PGDS and nephrin staining in podocytes exposed to vehicle (CTRL) or Adriamycin (ADR). **(L)** Statistical analysis of the mean fluorescence intensity of PGDS between vehicle and ADR podocytes. A two-tailed Mann–Whitney *U* test was used for each comparison. All differences with *P*< 0.05 are indicated, *P<0.05, **P<0.01, ***P<0.001, and ****P<0.0001. ns means no significant difference.

Understanding alterations in the molecular characteristics of kidney cells may help us to understand the downstream mechanisms of kidney injury in NS-IgAN. Considering the clinical characteristics of patients with NS-IgAN who have “nephrotic-range” proteinuria, we focused on the podocyte cluster that significantly expressed *NPHS2* and *FGF1*. Podocytes specifically expressed NPHS2 and FGF1 at both protein and transcriptional levels (**Figures 1E**, **4A, B**). Unfortunately, only two podocytes were captured in the SGC patient, and we did not include this group in the subsequent comparative analysis (**Supplementary Table 4**). We found that some genes showed a downward trend in terms of their expression by podocytes in the NS-IgAN group, including the canonical marker genes (*NPHS1* and *CLIC5*), the genes encoding membranous nephropathy-associated autoantigens (*PLA2R1* and *THSD7A*), and genes that have not been extensively investigated in podocytes, such as *PCOLCE2* and *PTGDS* (**Figure 4C**). Our data indicated that *CCL2* was highly expressed in the podocytes of NS-IgAN patients (**Figure 4C**). It has been reported that the expression of *CCL2* in podocytes is closely related to podocyte injury and proteinuria (24). In the adriamycin-induced mouse model of nephropathy, researchers found that CCL2 in the kidney may recruit the infiltration of inflammatory and pro-fibrotic bone marrow-derived cell populations through its receptor CCR2; furthermore, a deficiency of *Ccr2* in mice can ameliorate renal injury (17). We detected high expression levels of *CCL2* in NS-IgAN glomerular podocytes and significantly increased expression levels of *CCR2* in circulating monocytes (**Figure 2F**), thus indicating that crosstalk may occur in patients between podocytes and circulating monocytes through the CCL2-CCR2 ligand receptor. Furthermore, *CFH* was highly expressed in the podocytes of LGC (**Figure 4C**). Complement factor H (CFH) is one of the important circulating regulators of the alternative pathway, serves as an essential cofactor for complement factor I (CFI)-mediated C3b cleavage (25). Podocytes produce functionally active complement components, such as CFH; these could influence the local glomerular complement activation and regulation (26).

We also found that genes related to epithelial-to-mesenchymal transition (EMT) were significantly upregulated in NS-IgAN, including *TGFB1*, *CAV1*, *TAGLN*, and *COL1A1* (**Figure 4C**). We constructed an EMT gene set (**Supplementary Table 8**) and compared the gene set scores of podocytes between different groups. The EMT scores of podocytes in the NGC and LGC were significantly increased, while the EMT scores of podocytes in HU-IgAN did not change significantly (**Figure 4D**). EMT is an important feature of podocyte injury (27). Recent studies have found that podocytes in the urine of patients with NS also have EMT characteristics (28). Our results suggest that EMT is an important molecular feature of NS-IgAN podocytes. Interestingly, the expression of *PRSS23* was significantly elevated in NS-IgAN patients and to a greater degree than in HU-IgAN patients (**Figure 4C**). Bulk RNA-seq results also confirmed the reduced expression of *PRSS23* in various glomerular diseases (**Figure 4E**). These results suggest that a novel serine protease encoded by *PRSS23* may be related to podocyte injury.

### 3.6 The expression of *PTGDS* significantly decreased in damaged podocytes

By performing the scRNA-seq on kidney tissues, we found that *PTGDS* was only specifically expressed in the podocytes (**Figure 4F**). In addition, *PTGDS* was significantly downregulated in NS-IgAN podocytes (*P* < 0.0001) (**Figure 4G**). The glomerular transcriptome sequencing of various glomerular diseases, including membranous nephropathy (MN), focal segmental glomerulosclerosis (FSGS), and minimal change disease (MCD) confirmed that the transcriptional level of *PTGDS* was significantly decreased (**Figure 4H**). Hence, we reviewed the previously published scRNA-seq data of kidney tissues and found that *PTGDS* only expressed at high levels in human kidney podocytes (**Supplementary Figures 7A, B**). By using immunofluorescence and immunohistochemistry experiments, we were able to confirm the downregulation of PGDS (prostaglandin D2 synthase, encoded by *PTGDS*) in the glomeruli of patients with NS-IgAN (**Figures 4I, J**; **Supplementary Figures 8A, B**). The induction of injury in human immortalized podocytes *in vitro* (by applying doxorubicin) also led to a significant reduction in the expression levels of PGDS (**Figures 4K, L**; **Supplementary Figures 8C, D**). PGDS, also known as β-Trace protein (BTP), is an emerging novel marker for glomerular filtration rate (29). PGDS is produced *de novo* by both the glomeruli and LOHs in monkey kidneys (30), although the handling process of BTP in the kidney is uncertain. BTP is an eicosanoid that plays a role in a variety of important physiological processes, including vasodilation, inflammation, and adipogenesis (29). Our findings, and those of other researchers, indicate that *PTGDS* may represent a candidate marker gene for podocytes at the transcriptional level. *PTGDS* is expressed at high levels in normal podocytes and at far lower levels in injured podocytes.

### 3.7 Crosstalk between kidney cell types in NS-IgAN

**Figure 5:**
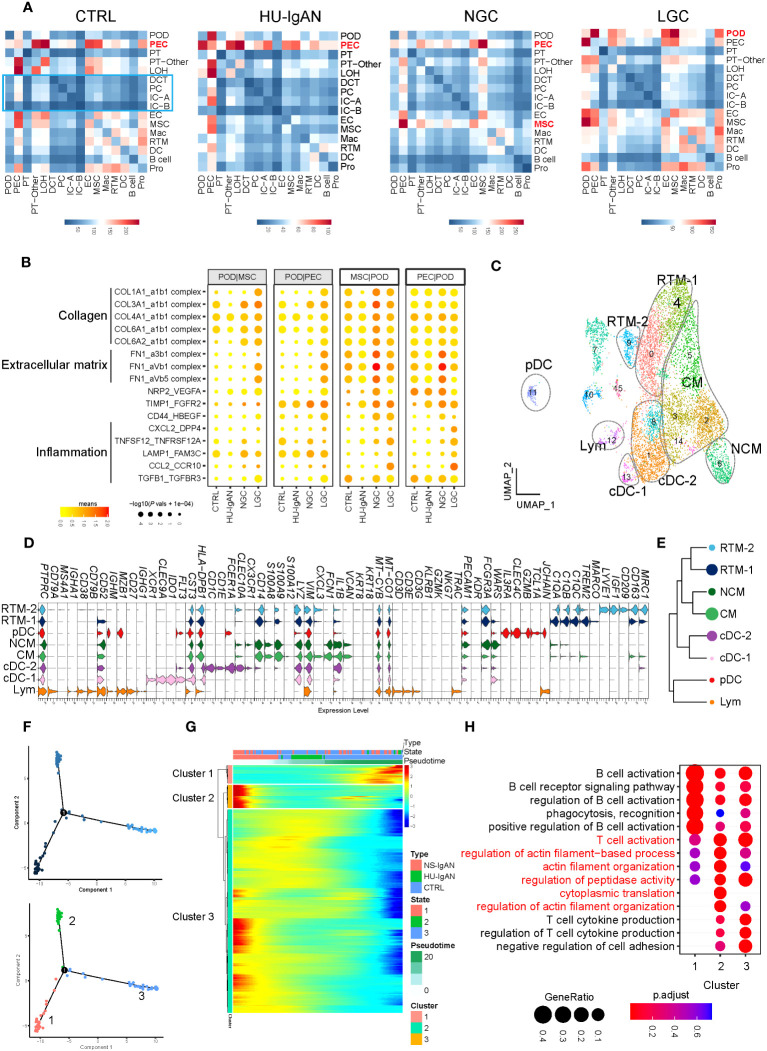
Molecular characterization of interactions between kidney cells and kidney immune cells. **(A)** Cell-to-cell crosstalk networks between kidney cells in CTRL, HU-IgAN, NGC, and LGC groups. **(B)** Bubble chart showing dysregulated cell-type specific interactions between mesenchymal stromal cells (MSCs) and podocytes (PODs) or parietal epithelial cells (PECs) and podocytes (PODs) in the CTRL, HU-IgAN, NGC, and LGC group. **(C)** 8 cell subtypes were visualized by UMAP plotting through re-clustering analysis of kidney immune cells. **(D)** Violin plots showing marker genes of 8 kidney immune cell subtypes. **(E)** Developmental tree analysis showed the relationship between different kidney immune cells. **(F)** Cell trajectory map of lymphocytes showing the pseudo-time (Top). Ordering single cells along a cell conversion trajectory using Monocle 2. Three states were identified based on their distribution in the cell trajectory map (Bottom). **(G)** Heatmap: each column represents one cell and each row represents the expression of one gene. Cells are ordered by Monocle-based pseudotime analysis and the color represents expression levels. **(H)** The GO enrichment function items of cluster1-3 in **(G)** are shown by the bubble diagram.

We investigated cell-to-cell communication between kidney cells by applying CellphoneDB. In the CTRL group, the cell types present in the proximal nephrons and kidney immune cells exhibited obvious intercellular communication, whereas distal convoluted tubules, intercalated cells and principal cells located in the distal nephron did not (**Figure 5A**). Our results suggest that the intercellular communication of the cells associated with the proximal nephron may be stronger than that of the cells associated with the distal nephron. In the CTRL and HU-IgAN groups, PECs may have served as the center for intercellular crosstalk in kidney cells (**Figure 5A**). In the NGC and LGC patients, the center of intercellular crosstalk was replaced by podocytes and mesenchymal stromal cells (MSCs) (**Figure 5A**). In an injury state, podocytes were affected by collagen-related, extracellular matrix-related, and inflammation-related signals from PECs and MSCs, such as COL3A1, FN1, and CCL2 (**Figure 5B**). These findings were consistent with previous findings related to IgAN (7, 8).

### 3.8 The molecular characteristics of renal lymphocytes

Kidney immune cells were re-clustered and defined into eight cell subsets by the expression of canonical marker genes (31) (**Figure 5C**, **Supplementary Table 9**). *C1QA*, *C1QB*, and *C1QC* are characteristically expressed in two subsets of tissue-resident macrophages (RTMs). Of these, RTM-1 expressed *LYVE1*, *IGF1*, and *CD209*, and RTM-2 expressed *TREM2* and *MARCO* (**Figure 5D**). Our results suggest that *FCN1* and *VCAN* may be specific markers of monocytes in the human kidney. Classical monocytes (CMs) in the kidney represented the main subset of monocytes and expressed *CXCL3*. In contrast, NCMs expressed *CX3CR1* (**Figure 5D**). *XCR1*, *CLEC9A*, and *IDO1* were expressed in Classical DC-1, and *CD1C* and *CD1E* were expressed in Classical DC-2. Plasmacytoid DC expressed *IL3RA*, *CLEC4C*, *GZMB*, and *TCL1A*. It is worth noting that cluster 12 simultaneously expressed the marker genes of B cells, including *CD79A* and *MZB1*, and also expressed the marker genes of T cells such as *CD3D*, *CD3E*, and *TRAC* (**Figure 5D**). These results indicated that this cluster may be composed of B lymphocytes and T lymphocytes; hence, we defined this cluster as lymphocytes. We performed a developmental analysis of eight groups of kidney immune cells and confirmed the difference between tissue-resident macrophages and monocytes (**Figure 5E**). Previous studies comprehensively demonstrated the molecular characteristics of myeloid cell subsets in the kidney (31–34); however, there was a lack of understanding of lymphocyte subsets in the kidney. We further performed pseudo-time analysis on lymphocytes and found that they were in three different states (states 1–3) in the kidneys (**Figure 5F**), with different molecular characteristics (clusters 1–3) (**Figure 5G**). Lymphocytes in state 3 significantly expressed genes related to cluster 1 (**Figure 5G**), which were related to B cell activation, phagocytosis, and recognition (**Figure 5H**). Lymphocytes in state 1 significantly expressed genes related to cluster 3 (**Figure 5G**). These results were related to the production of cytokines from T cells (**Figure 5H**). Surprisingly, state 2 lymphocytes significantly expressed genes related to cluster 2 (**Figure 5G**). These findings are related to T cell activation and actin filaments (**Figure 5H**). These results suggest that there may be a group of T cells with actin filament-related functions in the kidney. By applying a scRNA-seq technology platform, we were fortunate to capture immune cells in the kidney and provide a preliminary exploration of the molecular characteristics of lymphocytes. However, due to the limited number of immune cells captured from the kidney tissue involved in this study, we were not able to conduct further analysis.

### 3.9 DCs in circulation and kidney tissue expressed high levels of genes related to HLA

**Figure 6:**
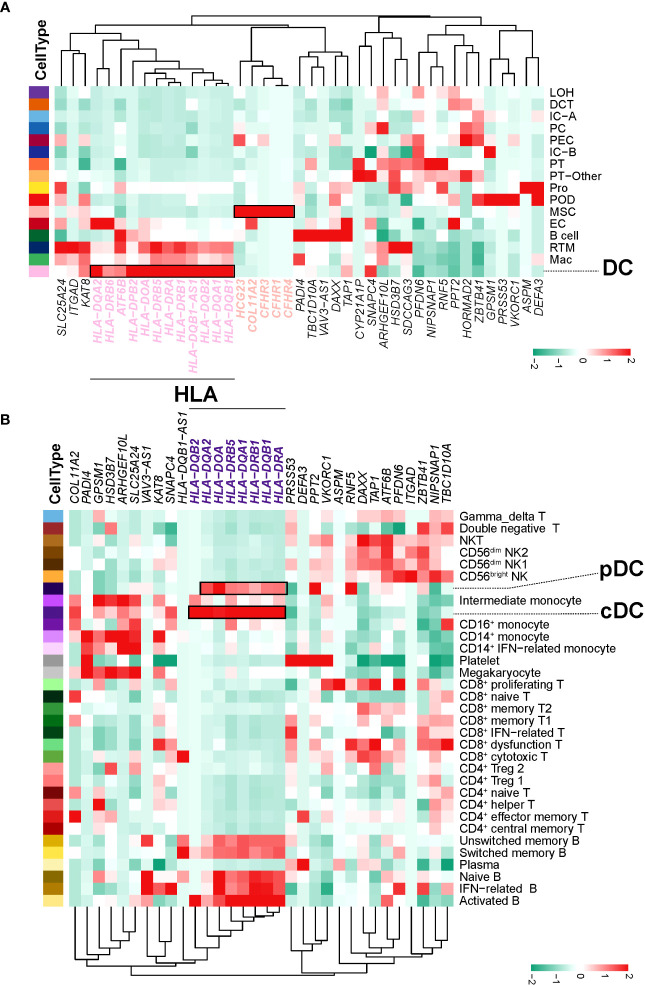
IgAN disease risk susceptibility genes expression. **(A)** The heatmap shows, for each IgAN susceptibility gene, the average expression over all cells in each kidney cell type. **(B)** The heatmap shows, for each IgAN susceptibility gene, the average expression over all cells in each PBMC cell subset.

Based on the SNPs detected in a recent GWAS meta-analysis of IgAN (18), we combined NephQTL and eQTL (cf.) to predict susceptibility genes that may be affected in different regions of the kidney tissue (**Supplementary Table 10**) and mapped these genes to kidney cells (**Figure 6A**) and PBMCs (**Figure 6B**). In the kidney tissue, renal resident macrophages, macrophages, and DC cells expressed high levels of genes related to HLA (**Figure 6A**). In circulation, multiple B cell subsets (except plasma cells), cDCs, and pDCs expressed high levels of genes related to HLA, while monocytes did not (**Figure 6B**). Previous studies had identified HLA molecules as the main disease related susceptibility loci for IgAN (18). Research evidence also indicated that DCs play key roles in the pathogenesis of IgAN (35). Our present data showed that DCs express high levels of HLA-related genes in both the circulation and kidney tissue. This indicated that attention needs to be paid to the changes in DC cells in the circulation and kidney tissue of NS-IgAN patients in future research (**Figures 6A, B**). The CFH-related genes *CFHR1*, *CFHR3*, and *CFHR4* were exclusively expressed in kidney MSCs (**Figure 6A**). We performed a re-clustering of the MSCs that significantly expressed *POSTN* and *ACTA2* (**Supplementary Table 4**; **Supplementary Figure 9A**). This analysis suggested that MSCs were a mixed subset composed of fibroblasts, myofibroblasts, vascular smooth muscle cells, pericytes, and mesangial cells (**Supplementary Figure 9B**). Unfortunately, in our study, mesangial cells could not be defined by canonical marker genes such as *PDGFRB*, *PIEZO2*, *ITGA8*, and *GATA3* (**Supplementary Figure 9B**).

## 4 Discussion

Glomerular diseases are still classified based on histological descriptions; however, these do not help capture the systemic mechanisms that drive the disease, nor are they suitable for target identification and drug development (36). Transcriptome sequencing at single-cell resolution, as represented by scRNA-seq and single-nucleus RNA-seq (snRNA-seq), is a powerful and new approach to unbiased analysis (37). However, it is challenging to create suitable designs for scRNA-seq experiments because each option requires the user to make informed decisions to obtain interpretable results (37, 38). Considering its low requirement for cell viability, we chose plate-based scRNA-seq technology to study core needle biopsies from the kidneys of patients (37, 39). We performed strict QC on the data and selected threshold settings based on the specificity of kidney cells (**Supplementary Table 2**). To identify mesangial cells, we performed a re-clustering of the MSCs (40). Our data suggested that MSCs may be composed of fibroblasts, myofibroblasts, vascular smooth muscle cells, pericytes, and mesangial cells (**Supplementary Figure 9**). As we were not able to annotate subgroups of cells by canonical marker genes, we were not able to perform downstream analysis of mesangial cells. Different methodological and technological platforms can lead to bias in capturing glomerular cell types; our results provide evidence for future single-cell research on mesangial-related glomerular diseases. Considering that immune cells in the kidney may play an important role in disease, we did not select snRNA-seq for our research to avoid losing molecular information related to immune cells (37) (**Figures 5C–H**).

Monocytes are innate immune cells that can be divided into three subsets based on the expression of CD14 and CD16 on the cell surface: classical, intermediate, and non-classical monocytes (14). Under inflammatory conditions, monocytes in the blood may migrate to the tissues and differentiate into mononuclear phagocytes in local regions of tissue (41). Previous research in the field of chronic kidney disease (CKD) has detected significant expansion of IMs (42), thus suggesting that changes in monocyte subsets may play an important role in CKD. Recent studies have found that CKD is associated with an increase in the number of unique proinflammatory IMs (HLA-DR^high^ IMs), as well as the migration of monocytes and endothelial adhesion abnormalities (43). Due to the limitation of renal biopsy in children with NS and the low incidence rate of NS-IgAN, we recruited children with NS as a validation patient cohort. The consistency of scRNA-seq data with the flow cytometry results of the validation cohort confirmed that IMs may be more abundant in NS-IgAN patients. Future studies on glomerular diseases need to pay specific attention to this group of IMs, particularly in terms of MHC class II analysis and chemokine signaling pathway functionality. In several human and experimental studies of proteinuria nephropathy, the expression of CCL2 was significantly localized in glomerular podocytes (17). It has also been confirmed that CCL2 does not only directly affect the actin cytoskeleton of podocytes (44), thus affecting the permeability of the slit diaphragm to albumin (45), but also causes indirect damage to podocytes by attracting macrophages and promoting inflammation (46). The expression of CCL2 in the kidney will recruit monocytes/macrophages expressing CCR2 into the circulation to be transported to the injury site and promote the differentiation of these myeloid cells toward the proinflammatory “M1” phenotype (47). Previous research suggested that the persistent infiltration of M1 macrophages and related inflammation can play a crucial pathogenic role in the development of podocyte dysfunction (48). The inhibition of CCR2 has been found to improve outcomes in animal models of FSGS (17), and several trials are currently underway to evaluate the impact of CCR2 inhibitors on FSGS patients (NCT03649152, NCT03536754, NCT03703908) (49). In addition, urinary CCL2 can be used as a biomarker for kidney inflammation (50) and these specific levels may be related to the extent of proteinuria (51). These data indicated that we cannot only gain an understanding of inflammatory progression in the kidney by detecting the urinary levels of CCL2. We may also be able to delay the chronic progression of the disease through new drugs targeting the CCL2-CCR2 ligand receptor pathway.

Podocyte damage is the key to the formation of proteinuria. The expression levels of EMT-related genes and *PRSS23* were significantly increased in podocytes from patients with NS-IgAN (**Figures 4C–E**). EMT is a functional and morphological alteration in podocyte injury (27, 28). The serine protease encoded by *PRSS23* activates protease-activated receptor 2, which is known to be associated with TGFβ1-induced podocyte injury in the rat model of doxorubicin nephropathy (52). The expression levels of canonical marker genes in the podocytes were significantly downregulated in NS-IgAN conditions, including those of *NPHS1*, *CLIC5*, and *MPP5* (**Figure 4C**). Our scRNA-seq results indicated that *PTGDS* was specifically expressed in podocytes and were significantly downregulated in NS-IgAN (**Figures 4G–J**). Unlike the remarkable specificity at the transcriptional level, immunohistochemistry and immunofluorescence results suggested that the specificity of PGDS at the protein level was limited (**Supplementary Figure 10**). BTP is a heterogeneous monomeric glycoprotein that is the consequence of post-translational N-glycosylation resulting in different glycoforms of varying molecular weight (53). The presence of multiple isomers certainly affects the measurement of BTP; the molecular structure of BTP produced by podocytes needs to be investigated further. Previous studies have suggested that in the early stages of diabetes nephropathy and CKD, the urinary levels of PGDS increased significantly (54). In the process of being excreted by the urine, some PGDS would be reabsorbed into the tubules and degraded by the lysosomes of tubule cells. We also noted that the staining intensity of PGDS was significantly reduced in the tubule cells of NS-IgAN patients (**Figure 4I**, **Supplementary Figure 10**). Whether this was due to the reduction of PGDS in podocytes or the reduced reabsorption capacity of renal tubules needs to be investigated in future research.

In our study, scRNA-seq of PBMCs and renal tissues were performed only on three pediatric patients with clinical manifestations of nephrotic syndrome. There was significant heterogeneity among the samples, and long-term cohort observations with larger sample sizes are required to truly elucidate the pathogenesis of NS-IgAN. Whether NS-IgAN is a combination of two diseases, is not discussed herein and needs to be further explored in future studies (4). Even though the findings of this study await future validation, they provide a rigorous framework for future research.

## Data availability statement

The original contributions presented in the study are publicly available. The datasets generated during and/or analyzed during the current study are available from the corresponding author on reasonable request.

## Ethics statement

Children’s Hospital of Chongqing Medical University approved this study (File Number: 2022 Research 124 and 2022 Research 35). The studies were conducted in accordance with the local legislation and institutional requirements. Written informed consent for participation in this study was provided by the participants’ legal guardians/next of kin. Ethical approval was not required for the studies on animals in accordance with the local legislation and institutional requirements because only commercially available established cell lines were used. Written informed consent was obtained from the individual(s), and minor(s)’ legal guardian/next of kin, for the publication of any potentially identifiable images or data included in this article.

## Author contributions

QC and HJ contributed equally to this work and share first authorship. QC conceptualized this project, interpreted the results of the analysis, prepared figures, and wrote the original paper. HJ enrolled and followed up with the patients, collected the samples, interpreted part of the results, and performed validation experiments. RD and JZ performed the single-cell transcriptome sequencing analyses and offered help with the bioinformatics analysis. JW, XF, and LP interpreted the results of the analysis, prepared figures, and wrote the manuscript. LL constructed the scRNA-seq library and performed the next-generation sequencing. XY, HC, AW, JJ, QY, XC, and XL prepared the single-cell suspensions of PBMCs and kidney biopsies. LS, GZ, and MW discussed the draft paper and critically reviewed the manuscript. HY and QL conceived and supervised the project and reviewed the manuscript. All authors read and commented on the manuscript. All authors contributed to the article and approved the submitted version.

## References

[1] Lai KN Tang SC Schena FP Novak J Tomino Y Fogo AB. IgA nephropathy. Nat Rev Dis Primers (2016) 2:16001. doi: 10.1038/nrdp.2016.1 27189177

[2] Nie S He W Huang T Liu D Wang G Geng J. The spectrum of biopsy-proven glomerular diseases among children in China: A national, cross-sectional survey. Clin J Am Soc Nephrol (2018) 13(7):1047–54. doi: 10.2215/cjn.11461017 PMC6032591 29915132

[3] Schena FP Rossini M Abbrescia DI Zaza G. The molecular mechanisms of inflammation and scarring in the kidneys of immunoglobulin A nephropathy : Gene involvement in the mechanisms of inflammation and scarring in kidney biopsy of IgAN patients. Semin Immunopathol (2021) 43(5):691–705. doi: 10.1007/s00281-021-00891-8 34674036 PMC8551145

[4] Shima Y Nakanishi K Sato M Hama T Mukaiyama H Togawa H. IgA nephropathy with presentation of nephrotic syndrome at onset in children. Pediatr Nephrol (2017) 32(3):457–65. doi: 10.1007/s00467-016-3502-6 27714465

[5] Kim JK Kim JH Lee SC Kang EW Chang TI Moon SJ. Clinical features and outcomes of IgA nephropathy with nephrotic syndrome. Clin J Am Soc Nephrol (2012) 7(3):427–36. doi: 10.2215/CJN.04820511 PMC3302681 22223610

[6] Pattrapornpisut P Avila-Casado C Reich HN. IgA nephropathy: core curriculum 2021. Am J Kidney Dis (2021) 78(3):429–41. doi: 10.1053/j.ajkd.2021.01.024 34247883

[7] Lemley KV Lafayette RA Safai M Derby G Blouch K Squarer A. Podocytopenia and disease severity in IgA nephropathy. Kidney Int (2002) 61(4):1475–85. doi: 10.1046/j.1523-1755.2002.00269.x 11918755

[8] Lai KN Tang SC Guh JY Chuang TD Lam MF Chan LY. Polymeric IgA1 from patients with IgA nephropathy upregulates transforming growth factor-beta synthesis and signal transduction in human mesangial cells *via* the renin-angiotensin system. J Am Soc Nephrol (2003) 14(12):3127–37. doi: 10.1097/01.asn.0000095639.56212.bf 14638911

[9] Stewart BJ Ferdinand JR Clatworthy MR. Using single-cell technologies to map the human immune system - implications for nephrology. Nat Rev Nephrol (2020) 16(2):112–28. doi: 10.1038/s41581-019-0227-3 31831877

[10] Zheng Y Lu P Deng Y Wen L Wang Y Ma X. Single-cell transcriptomics reveal immune mechanisms of the onset and progression of igA nephropathy. Cell Rep (2020) 33(12):108525. doi: 10.1016/j.celrep.2020.108525 33357427

[11] Tang R Meng T Lin W Shen C Ooi JD Eggenhuizen PJ. A partial picture of the single-cell transcriptomics of human igA nephropathy. Front Immunol (2021) 12:645988. doi: 10.3389/fimmu.2021.645988 33936064 PMC8085501

[12] Zeng H Wang L Li J Luo S Han Q Su F. Single-cell RNA-sequencing reveals distinct immune cell subsets and signaling pathways in IgA nephropathy. Cell Biosci (2021) 11(1):203. doi: 10.1186/s13578-021-00706-1 34895340 PMC8665497

[13] Wu J Zheng C Wang X Yun S Zhao Y Liu L. MicroRNA-30 family members regulate calcium/calcineurin signaling in podocytes. J Clin Invest (2015) 125(11):4091–106. doi: 10.1172/JCI81061 PMC4639992 26436650

[14] Ziegler-Heitbrock L Ancuta P Crowe S Dalod M Grau V Hart DN. Nomenclature of monocytes and dendritic cells in blood. Blood (2010) 116(16):e74–80. doi: 10.1182/blood-2010-02-258558 20628149

[15] Cox SN Serino G Sallustio F Blasi A Rossini M Pesce F. Altered monocyte expression and expansion of non-classical monocyte subset in IgA nephropathy patients. Nephrol Dial Transpl (2015) 30(7):1122–232. doi: 10.1093/ndt/gfv017 25770168

[16] Franca CN Izar MCO Hortencio MNS do Amaral JB Ferreira CES Tuleta ID. Monocyte subtypes and the CCR2 chemokine receptor in cardiovascular disease. Clin Sci (Lond) (2017) 131(12):1215–24. doi: 10.1042/CS20170009 28566450

[17] Wilkening A Krappe J Muhe AM Lindenmeyer MT Eltrich N Luckow B. C-C chemokine receptor type 2 mediates glomerular injury and interstitial fibrosis in focal segmental glomerulosclerosis. Nephrol Dial Transpl (2020) 35(2):227–39. doi: 10.1093/ndt/gfy380 30597038

[18] Li M Wang L Shi DC Foo JN Zhong Z Khor CC. Genome-wide meta-analysis identifies three novel susceptibility loci and reveals ethnic heterogeneity of genetic susceptibility for IgA nephropathy. J Am Soc Nephrol (2020) 31(12):2949–63. doi: 10.1681/ASN.2019080799 PMC7790208 32912934

[19] Huang H Sun W Liang Y Peng Y Long XD Liu Z. CD4 (+)CD 25 (+)Treg cells and IgA nephropathy patients with tonsillectomy: a clinical and pathological study. Int Urol Nephrol (2014) 46(12):2361–9. doi: 10.1007/s11255-014-0851-6 25281312

[20] Wing JB Tanaka A Sakaguchi S. Human FOXP3(+) regulatory T cell heterogeneity and function in autoimmunity and cancer. Immunity (2019) 50(2):302–16. doi: 10.1016/j.immuni.2019.01.020 30784578

[21] Zemmour D Charbonnier LM Leon J Six E Keles S Delville M. Single-cell analysis of FOXP3 deficiencies in humans and mice unmasks intrinsic and extrinsic CD4(+) T cell perturbations. Nat Immunol (2021) 22(5):607–19. doi: 10.1038/s41590-021-00910-8 PMC8173714 33833438

[22] Sakai R Ito M Komai K Iizuka-Koga M Matsuo K Nakayama T. Kidney GATA3(+) regulatory T cells play roles in the convalescence stage after antibody-mediated renal injury. Cell Mol Immunol (2021) 18(5):1249–61. doi: 10.1038/s41423-020-00547-x PMC8093306 32917984

[23] Ju W Greene CS Eichinger F Nair V Hodgin JB Bitzer M. Defining cell-type specificity at the transcriptional level in human disease. Genome Res (2013) 23(11):1862–73. doi: 10.1101/gr.155697.113 PMC3814886 23950145

[24] Ren J Xu Y Lu X Wang L Ide S Hall G. Twist1 in podocytes ameliorates podocyte injury and proteinuria by limiting CCL2-dependent macrophage infiltration. JCI Insight (2021) 6(15):e148109. doi: 10.1172/jci.insight.148109 34369383 PMC8410065

[25] Pickering MC Cook HT. Translational mini-review series on complement factor H: renal diseases associated with complement factor H: novel insights from humans and animals. Clin Exp Immunol (2008) 151(2):210–30. doi: 10.1111/j.1365-2249.2007.03574.x PMC2276951 18190458

[26] Muhlig AK Keir LS Abt JC Heidelbach HS Horton R Welsh GI. Podocytes produce and secrete functional complement C3 and complement factor H. Front Immunol (2020) 11:1833. doi: 10.3389/fimmu.2020.01833 32922395 PMC7457071

[27] Li Y Kang YS Dai C Kiss LP Wen X Liu Y. Epithelial-to-mesenchymal transition is a potential pathway leading to podocyte dysfunction and proteinuria. Am J Pathol (2008) 172(2):299–308. doi: 10.2353/ajpath.2008.070057 18202193 PMC2312375

[28] Latt KZ Heymann J Jessee JH Rosenberg AZ Berthier CC Arazi A. Urine single-cell RNA sequencing in focal segmental glomerulosclerosis reveals inflammatory signatures. Kidney Int Rep (2022) 7(2):289–304. doi: 10.1016/j.ekir.2021.11.005 35155868 PMC8821042

[29] White CA Ghazan-Shahi S Adams MA. beta-Trace protein: a marker of GFR and other biological pathways. Am J Kidney Dis (2015) 65(1):131–46. doi: 10.1053/j.ajkd.2014.06.038 25446025

[30] Nagata N Fujimori K Okazaki I Oda H Eguchi N Uehara Y. *De novo* synthesis, uptake and proteolytic processing of lipocalin-type prostaglandin D synthase, beta-trace, in the kidneys. FEBS J (2009) 276(23):7146–58. doi: 10.1111/j.1742-4658.2009.07426.x 19878301

[31] Stewart BJ Ferdinand JR Young MD Mitchell TJ Loudon KW Riding AM. Spatiotemporal immune zonation of the human kidney. Science (2019) 365(6460):1461–6. doi: 10.1126/science.aat5031 PMC7343525 31604275

[32] Zimmerman KA Bentley MR Lever JM Li Z Crossman DK Song CJ. Single-cell RNA sequencing identifies candidate renal resident macrophage gene expression signatures across species. J Am Soc Nephrol (2019) 30(5):767–81. doi: 10.1681/ASN.2018090931 PMC6493978 30948627

[33] McEvoy CM Murphy JM Zhang L Clotet-Freixas S Mathews JA An J. Single-cell profiling of healthy human kidney reveals features of sex-based transcriptional programs and tissue-specific immunity. Nat Commun (2022) 13(1):7634. doi: 10.1038/s41467-022-35297-z 36496458 PMC9741629

[34] Conway BR O'Sullivan ED Cairns C O'Sullivan J Simpson DJ Salzano A. Kidney single-cell atlas reveals myeloid heterogeneity in progression and regression of kidney disease. J Am Soc Nephrol (2020) 31(12):2833–54. doi: 10.1681/asn.2020060806 PMC7790206 32978267

[35] Takechi H Oda T Hotta O Yamamoto K Oshima N Matsunobu T. Clinical and immunological implications of increase in CD208+ dendritic cells in tonsils of patients with immunoglobulin A nephropathy. Nephrol Dial Transpl (2013) 28(12):3004–13. doi: 10.1093/ndt/gft399 PMC3843345 24081865

[36] Kalantar-Zadeh K Jafar TH Nitsch D Neuen BL Perkovic V. Chronic kidney disease. Lancet (2021) 398(10302):786–802. doi: 10.1016/s0140-6736(21)00519-5 34175022

[37] Deleersnijder D Callemeyn J Arijs I Naesens M Van Craenenbroeck AH Lambrechts D. Current methodological challenges of single-cell and single-nucleus RNA-sequencing in glomerular diseases. J Am Soc Nephrol (2021) 32(8):1838–52. doi: 10.1681/ASN.2021020157 PMC8455274 34140401

[38] Lafzi A Moutinho C Picelli S Heyn H. Tutorial: guidelines for the experimental design of single-cell RNA sequencing studies. Nat Protoc (2018) 13(12):2742–57. doi: 10.1038/s41596-018-0073-y 30446749

[39] Xu J Shen C Lin W Meng T Ooi JD Eggenhuizen PJ. Single-cell profiling reveals transcriptional signatures and cell-cell crosstalk in anti-PLA2R positive idiopathic membranous nephropathy patients. Front Immunol (2021) 12:683330. doi: 10.3389/fimmu.2021.683330 34135910 PMC8202011

[40] Avraham S Korin B Chung JJ Oxburgh L Shaw AS. The Mesangial cell - the glomerular stromal cell. Nat Rev Nephrol (2021) 17(12):855–64. doi: 10.1038/s41581-021-00474-8 34508249

[41] Narasimhan PB Marcovecchio P Hamers AAJ Hedrick CC. Nonclassical monocytes in health and disease. Annu Rev Immunol (2019) 37:439–56. doi: 10.1146/annurev-immunol-042617-053119 31026415

[42] Naicker SD Cormican S Griffin TP Maretto S Martin WP Ferguson JP. Chronic kidney disease severity is associated with selective expansion of a distinctive intermediate monocyte subpopulation. Front Immunol (2018) 9:2845. doi: 10.3389/fimmu.2018.02845 30619252 PMC6302774

[43] Cormican S Negi N Naicker SD Islam MN Fazekas B Power R. Chronic kidney disease is characterized by expansion of a distinct proinflammatory intermediate monocyte subtype and by increased monocyte adhesion to endothelial cells. J Am Soc Nephrol (2023) 34(5):793–808. doi: 10.1681/ASN.0000000000000083 PMC10125648 36799882

[44] Lee EY Chung CH Khoury CC Yeo TK Pyagay PE Wang A. The monocyte chemoattractant protein-1/CCR2 loop, inducible by TGF-beta, increases podocyte motility and albumin permeability. Am J Physiol Renal Physiol (2009) 297(1):F85–94. doi: 10.1152/ajprenal.90642.2008 PMC2711714 19420107

[45] Burt D Salvidio G Tarabra E Barutta F Pinach S Dentelli P. The monocyte chemoattractant protein-1/cognate CC chemokine receptor 2 system affects cell motility in cultured human podocytes. Am J Pathol (2007) 171(6):1789–99. doi: 10.2353/ajpath.2007.070398 PMC2111103 18055544

[46] Bruggeman LA Drawz PE Kahoud N Lin K Barisoni L Nelson PJ. TNFR2 interposes the proliferative and NF-kappaB-mediated inflammatory response by podocytes to TNF-alpha. Lab Invest (2011) 91(3):413–25. doi: 10.1038/labinvest.2010.199 PMC3075956 21221075

[47] Fujinaka H Yamamoto T Takeya M Feng L Kawasaki K Yaoita E. Suppression of anti-glomerular basement membrane nephritis by administration of anti-monocyte chemoattractant protein-1 antibody in WKY rats. J Am Soc Nephrol (1997) 8(7):1174–8. doi: 10.1681/ASN.V871174 9219168

[48] Bell RMB Denby L. Myeloid heterogeneity in kidney disease as revealed through single-cell RNA sequencing. Kidney360 (2021) 2(11):1844–51. doi: 10.34067/KID.0003682021 PMC8785845 35372996

[49] De Vriese AS Wetzels JF Glassock RJ Sethi S Fervenza FC. Therapeutic trials in adult FSGS: lessons learned and the road forward. Nat Rev Nephrol (2021) 17(9):619–30. doi: 10.1038/s41581-021-00427-1 PMC8136112 34017116

[50] Puthumana J Thiessen-Philbrook H Xu L Coca SG Garg AX Himmelfarb J. Biomarkers of inflammation and repair in kidney disease progression. J Clin Invest (2021) 131(3):e139927. doi: 10.1172/JCI139927 33290282 PMC7843225

[51] Eardley KS Zehnder D Quinkler M Lepenies J Bates RL Savage CO. The relationship between albuminuria, MCP-1/CCL2, and interstitial macrophages in chronic kidney disease. Kidney Int (2006) 69(7):1189–97. doi: 10.1038/sj.ki.5000212 16609683

[52] Wang Y He Y Wang M Lv P Liu J Wang J. Role of protease-activated receptor 2 in regulating focal segmental glomerulosclerosis. Cell Physiol Biochem (2017) 41(3):1147–55. doi: 10.1159/000464121 28245472

[53] Urade Y Hayaishi O. Biochemical, structural, genetic, physiological, and pathophysiological features of lipocalin-type prostaglandin D synthase. Biochim Biophys Acta (2000) 1482(1-2):259–71. doi: 10.1016/s0167-4838(00)00161-8 11058767

[54] Oda H Shiina Y Seiki K Sato N Eguchi N Urade Y. Development and evaluation of a practical ELISA for human urinary lipocalin-type prostaglandin D synthase. Clin Chem (2002) 48(9):1445–53. 12194921

