# Supplementary material for: Cell-type-specific molecular characterization of cells from circulation and kidney in IgA nephropathy with nephrotic syndrome
Source: Front Immunol. 2023 Oct 16;14:1231937. doi: 10.3389/fimmu.2023.1231937 (PMC10613708; doi:10.3389/fimmu.2023.1231937)
Supplement: Supplementary file 1 [file DataSheet_1.pdf]

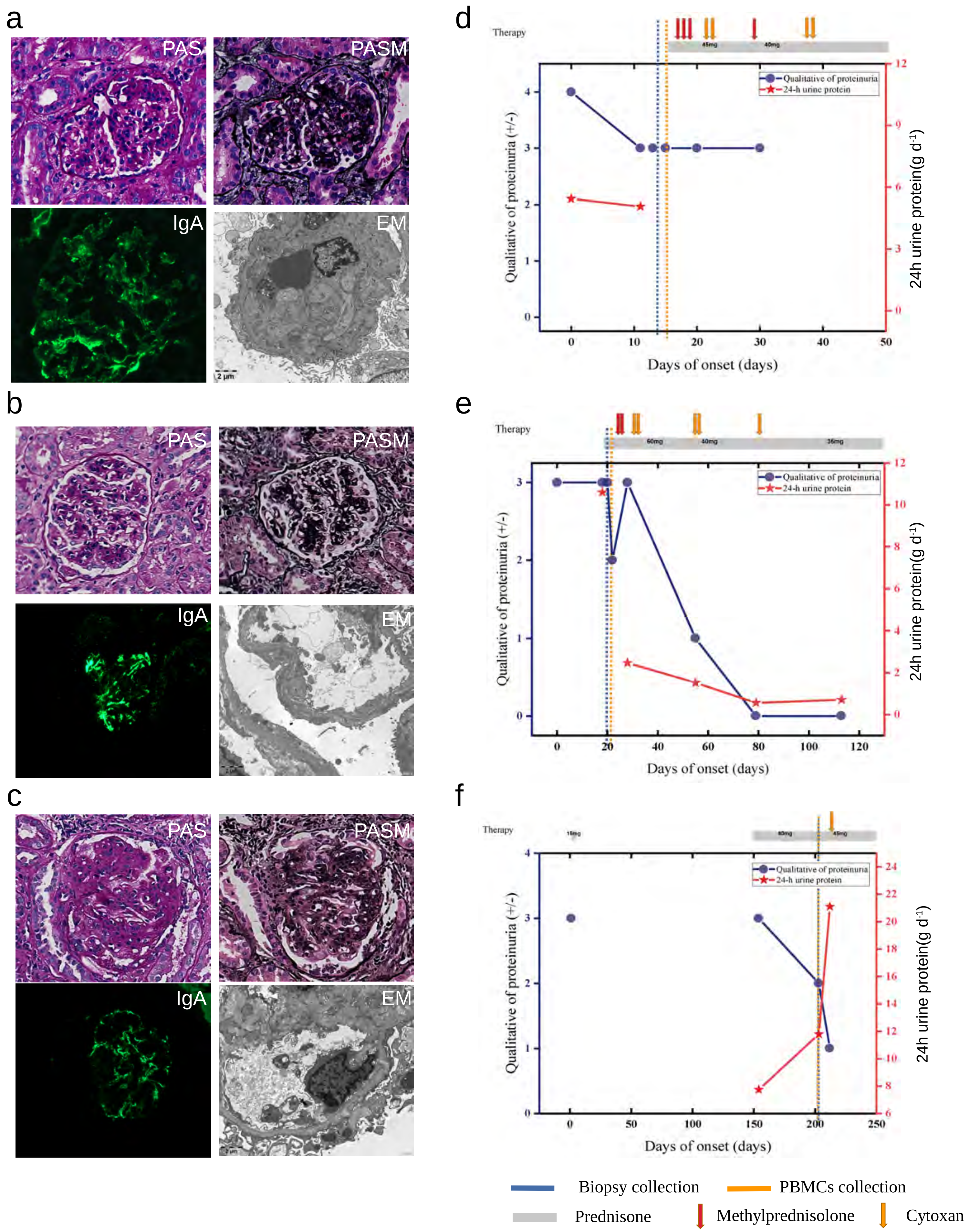

Supplementary figure 1: Information on the diagnosis and treatment process of NS-IgAN patients. (a-c) Pathological diagnosis images of NGC (a), SGC (b), and LGC (c). For each patient, the four pathological images show periodic acid-Schiff (PAS) staining, periodic acid-silver methenamine (PASM) staining, IgA immunofluorescence and electron microscopy (EM). (d-f)Schematic diagram of the diagnosis and treatment process of NGC (d), SGC (e), and LGC (f) patients. The abscissa represents the number of days since the onset of illness, and the ordinate on the left represents qualitative value of quantitative value and the right represents the 24-hour urine protein quantitative value at the corresponding time point.

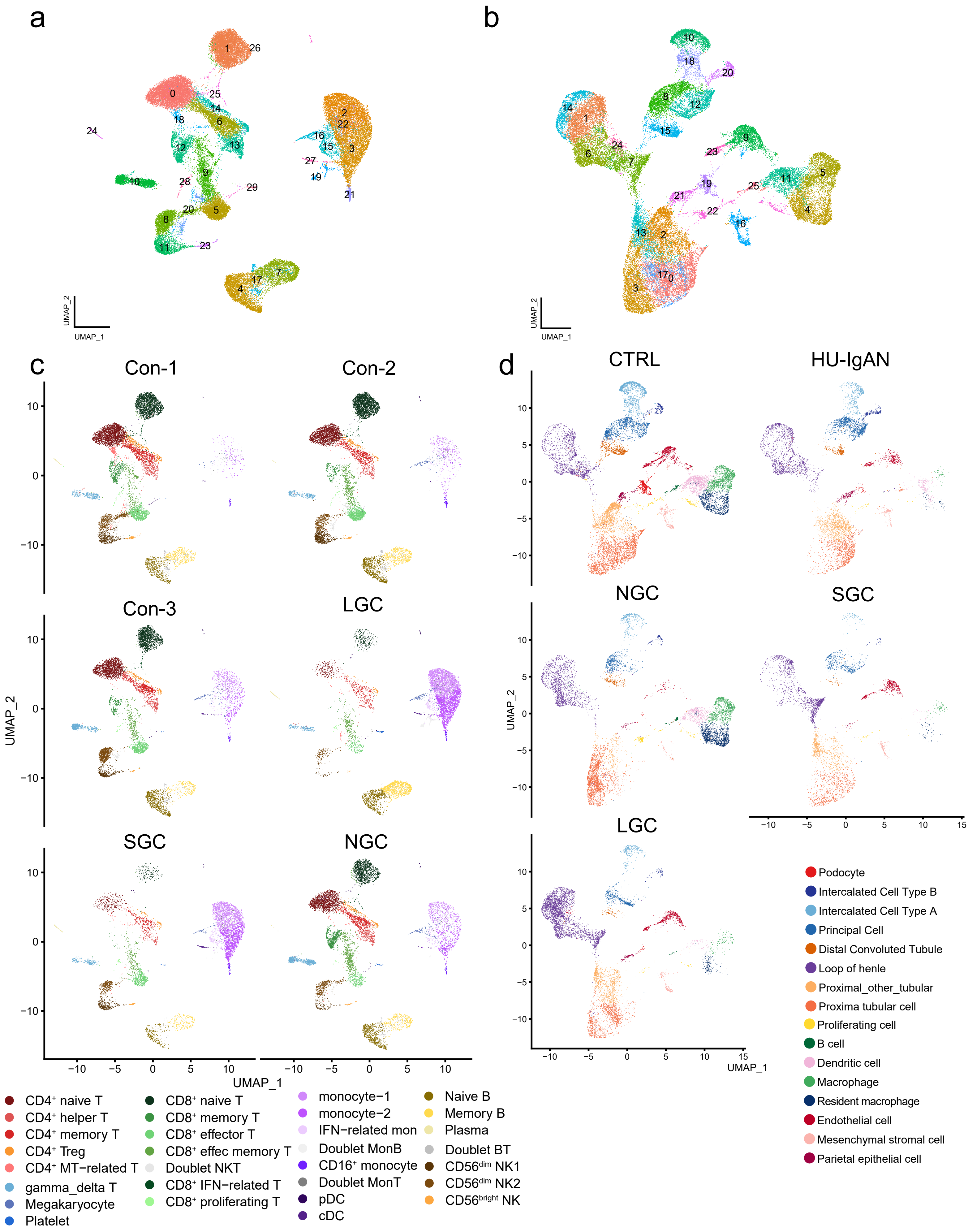

Supplementary figure 2: Two-dimensional UMAP visualization of 30 clusters in PBMCs (a) and 26 clusters in kidney cells (b) for each samples (c, d).

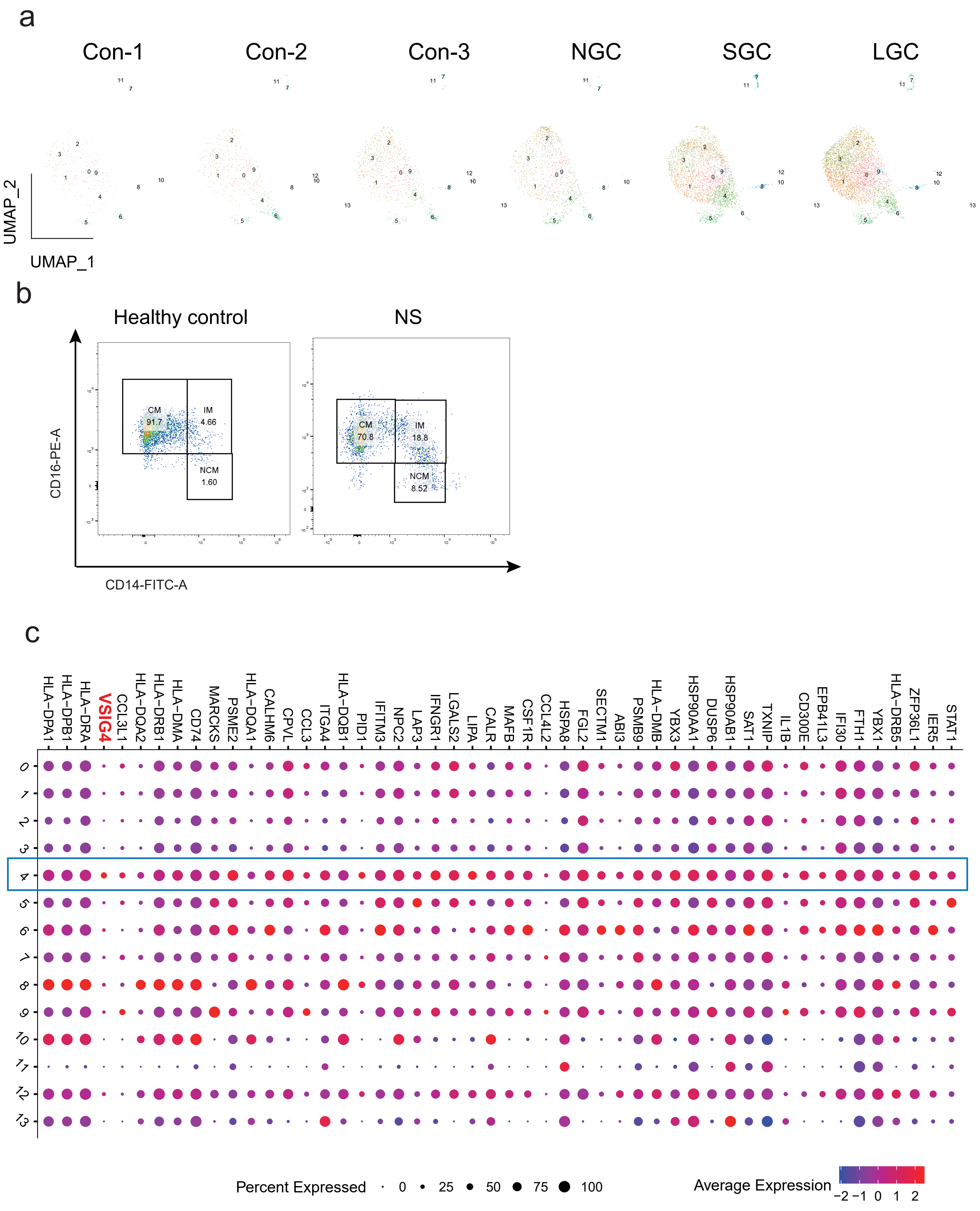

Supplementary figure 3: Changes of myeloid cell subsets in NS-IgAN. (a) Proportion of myeloid cells subsets separated by samples. (b) Gating strategy of monocyte subsets in human peripheral blood analyzed by flow cytometry, the left is healthy controls, and right is NS patients. (c) Dot plot of select average gene expression values (log scale) and percentage of expressing these genes within cluster 4 (IMs) for top 50 signature gene.

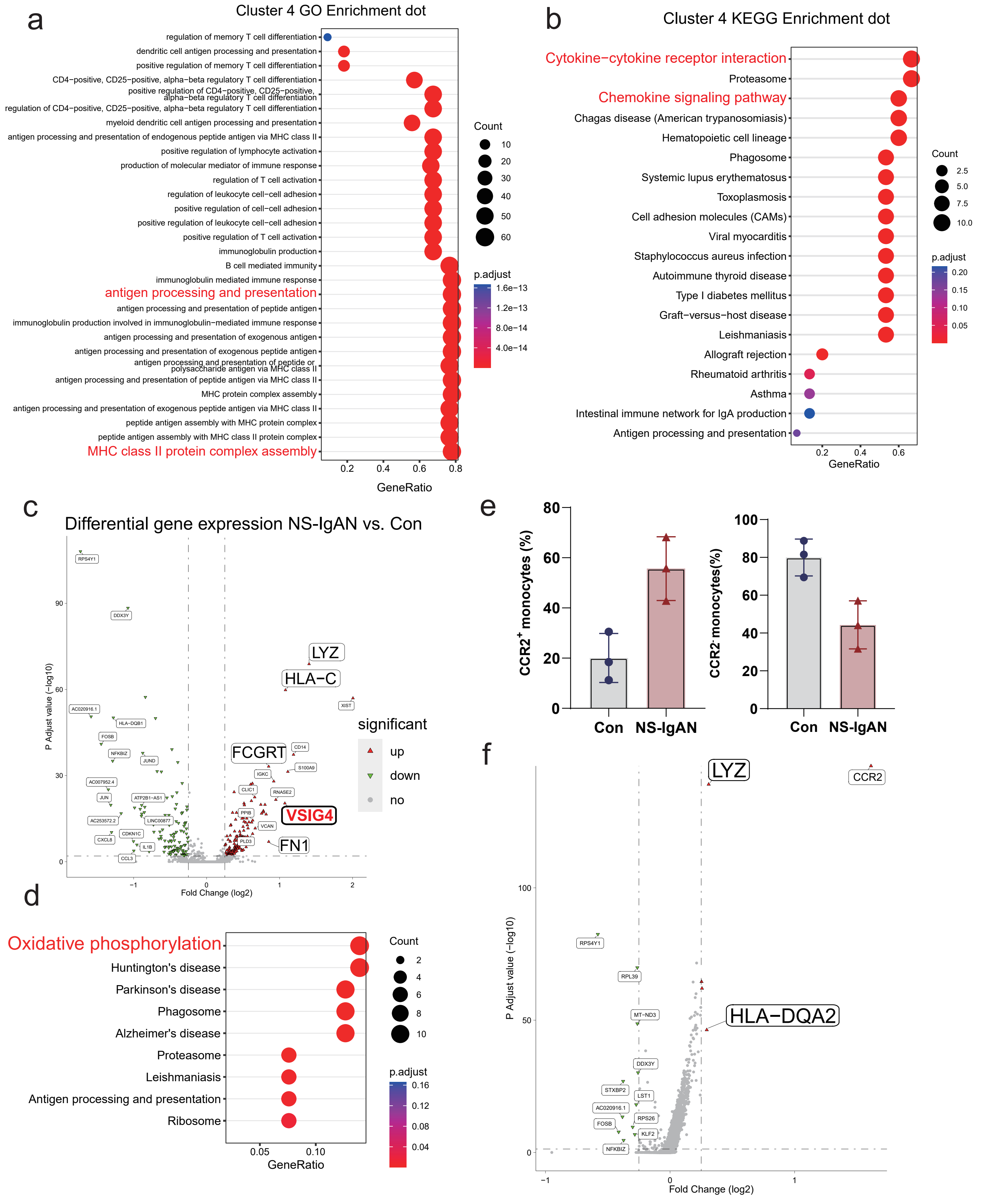

Supplementary figure 4: The functional feature of IMs and the changes of CCR2$^+$ monocytes. GO (a) and KEGG (b) analysis via comparison of IMs vs. others. Terms are labeled with name, and sorted by $-\log_{10}$(P) value. A darker color indicates a smaller P value. Interesting terms are labeled in red. (c) Volcano plot showing differentially expressed genes (DEGs) in IMs between NS-IgAN and Con. (d) KEGG enrichment analysis of UP-DEGs of IMs in NS-IgAN than in Con. (e) The percentage of CCR2$^+$ monocytes (left) and CCR2$^-$ monocytes (right) in all monocytes. (f) Volcano plot showing DEGs between CCR2$^+$ monocytes and CCR2$^-$ monocytes.

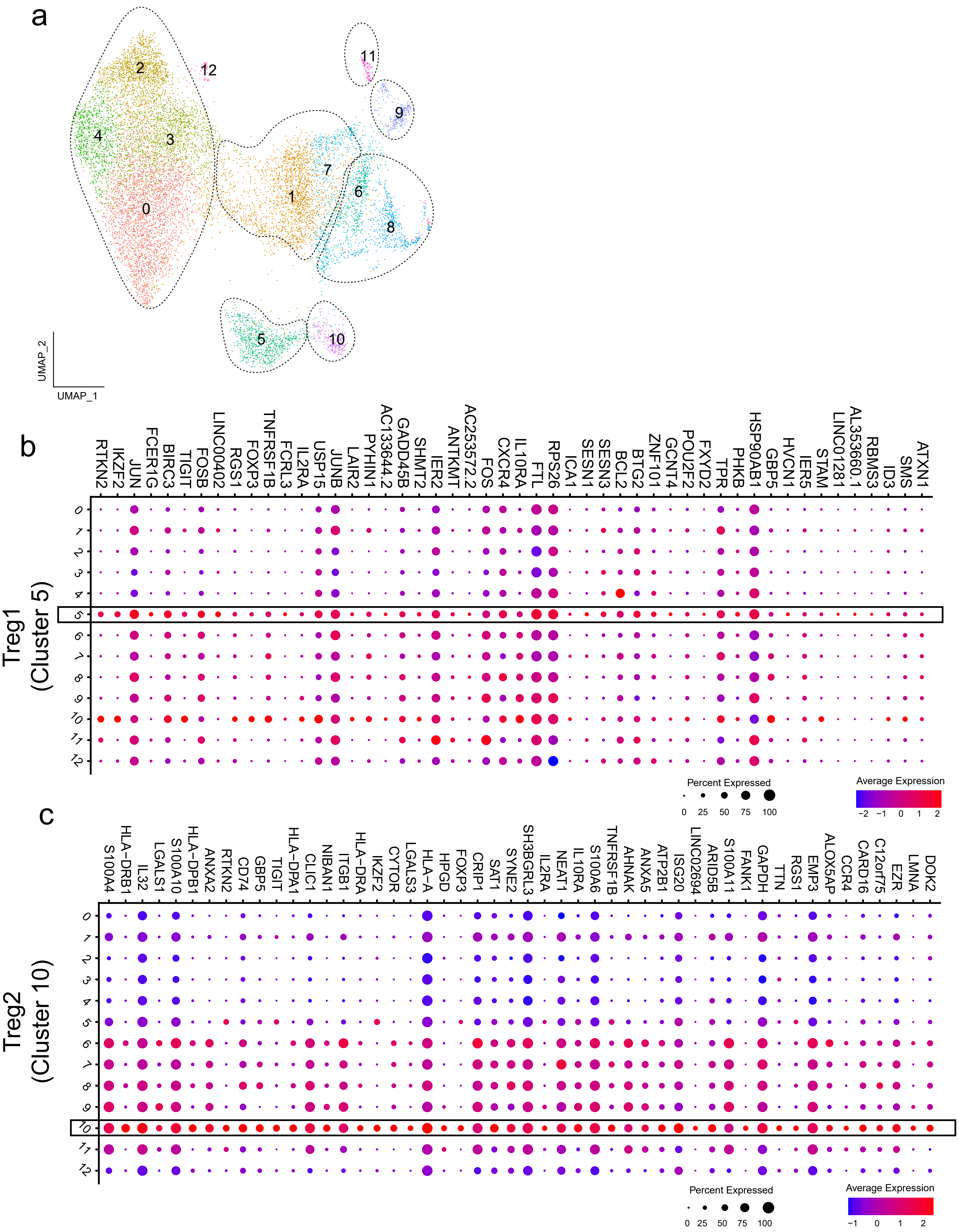

Supplementary figure 5: The molecular expression of CD4+ T cell subsets. (a) UMAP visualization of 13 clusters in CD4+ T cells. Dot plot of select average gene expression values (log scale) and percentage of expressing these genes within cluster 5 (b) and cluster 10 (c) for top 50 signature gene.

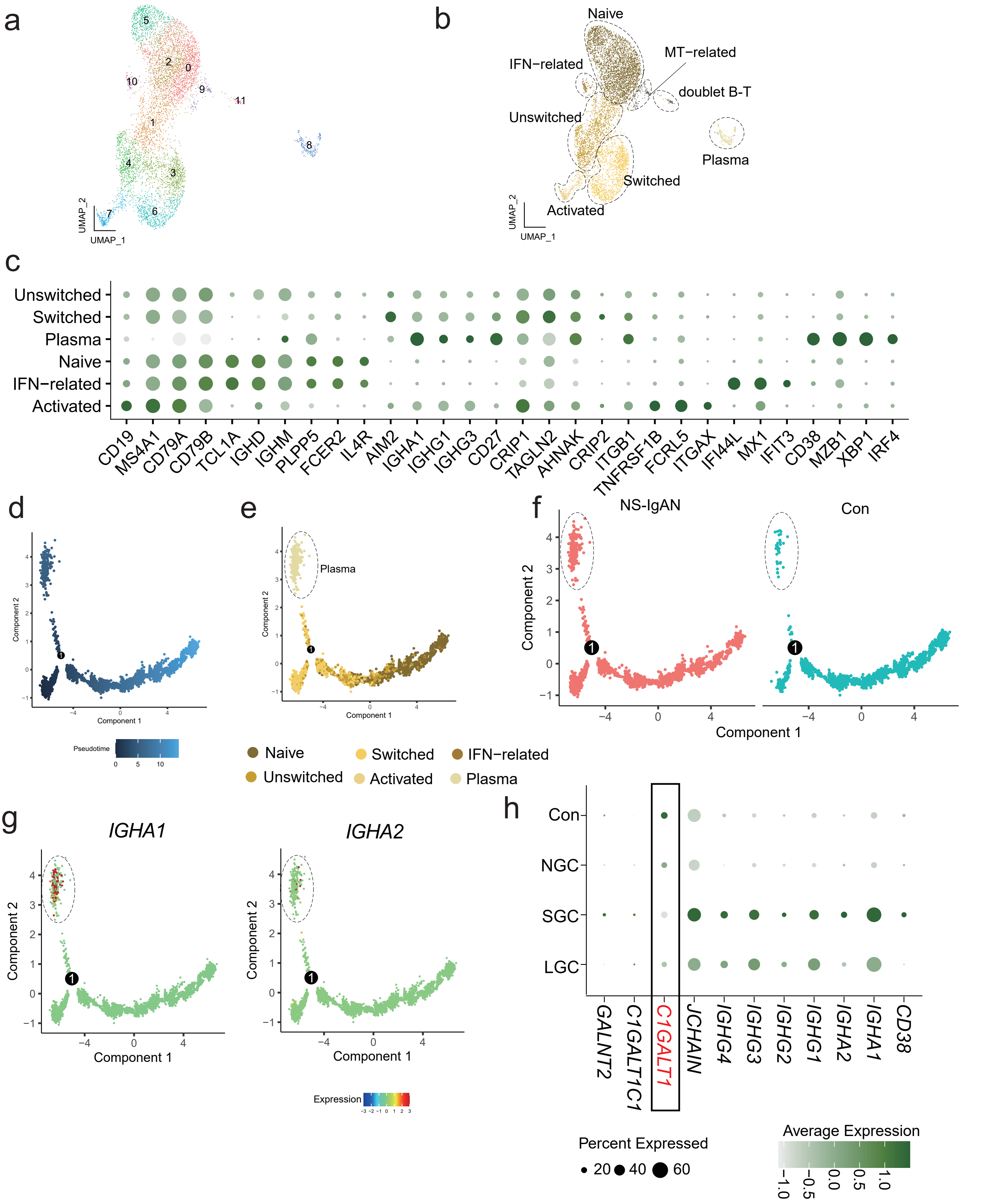

Supplementary figure 6: Molecular characterization of B cells in NS-IgAN. UMAP visualization of 13 clusters (a) and 6 cell types in B cells. (c) Dot plot of marker genes in 6 subsets of B cells. (d) Cell trajectory map of B cells showing the pseudo-time. Six cell types (e), NS-IgAN and Con (f) of B cells were identified based on their distribution in the cell trajectory map. (g) Cell trajectory map of B cells showing the expression level of IGHA1 and IGHA2. (h) Dot plots showing function-related genes in B cells.

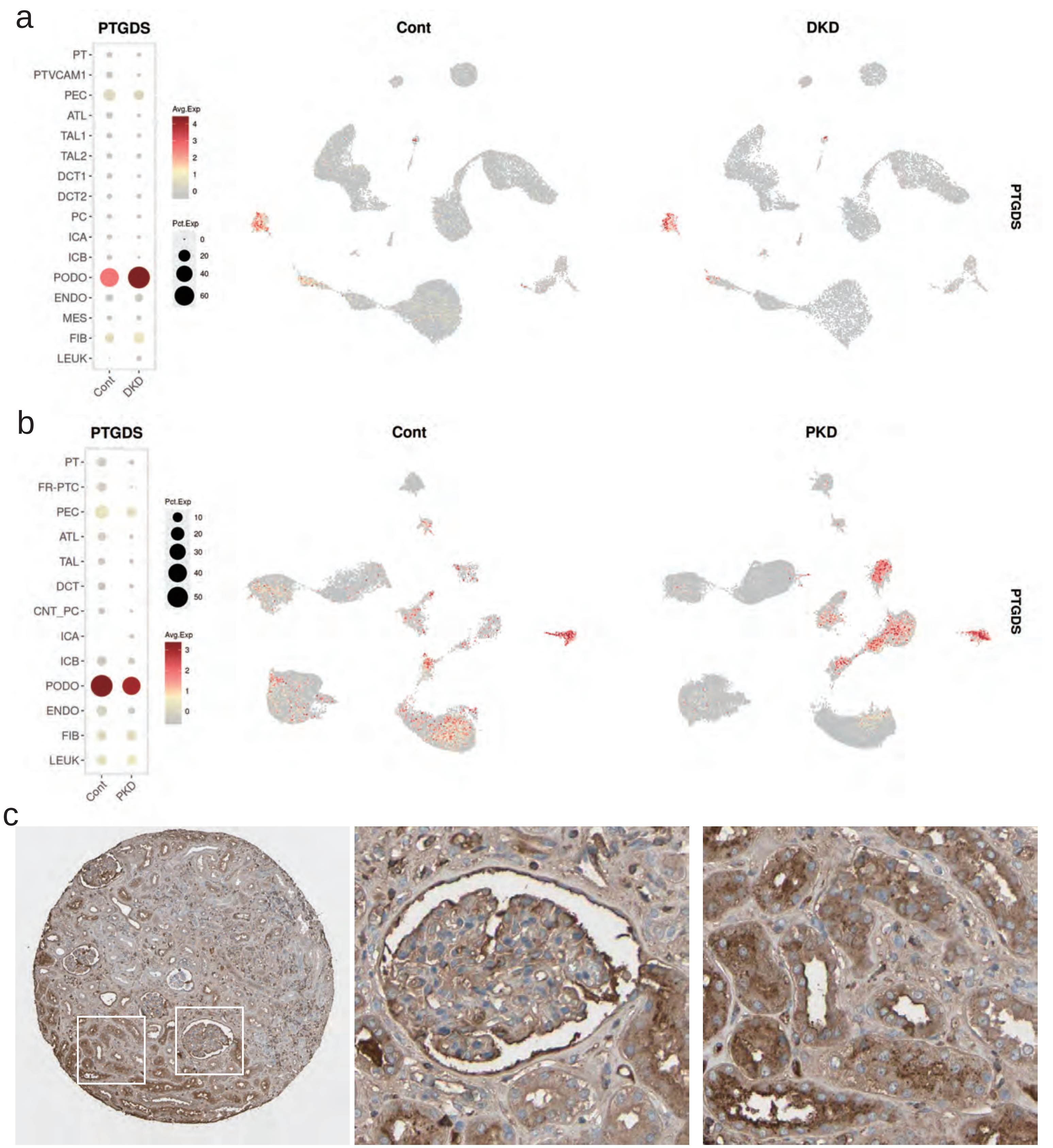

Supplementary figure 7: Expression of PTGDS in the kidney in snRNA-seq (http://humphreyslab.com/SingleCell/).
(a) Cell distribution of PTGDS in snRNA-seq of kidney tissues from healthy human (Cont) and diabetic kidney disease (DKD) by Wilson and Wilson and Muto et al. (2022)[1]. (b) Cell distribution of PTGDS in snRNA-seq of kidney tissues from healthy human (Cont) and polycystic kidney disease (PKD) by Muto et al. (2022)[2].
(c) Immunohistochemistry showing expression of PGDS in glomeruli or tubules in normal human kidney from Human Protein Atlas (https://www.proteinatlas.org/).

Reference:

1.Wilson PC, Muto Y, Wu H, Karihaloo A, Waikar SS, Humphreys BD. Multimodal single cell sequencing implicates chromatin accessibility and genetic background in diabetic kidney disease progression. Nat Commun 2022;13:5253.
2.Muto Y, Dixon EE, Yoshimura Y, et al. Defining cellular complexity in human autosomal dominant polycystic kidney disease by multimodal single cell analysis. Nat Commun 2022;13:6497.

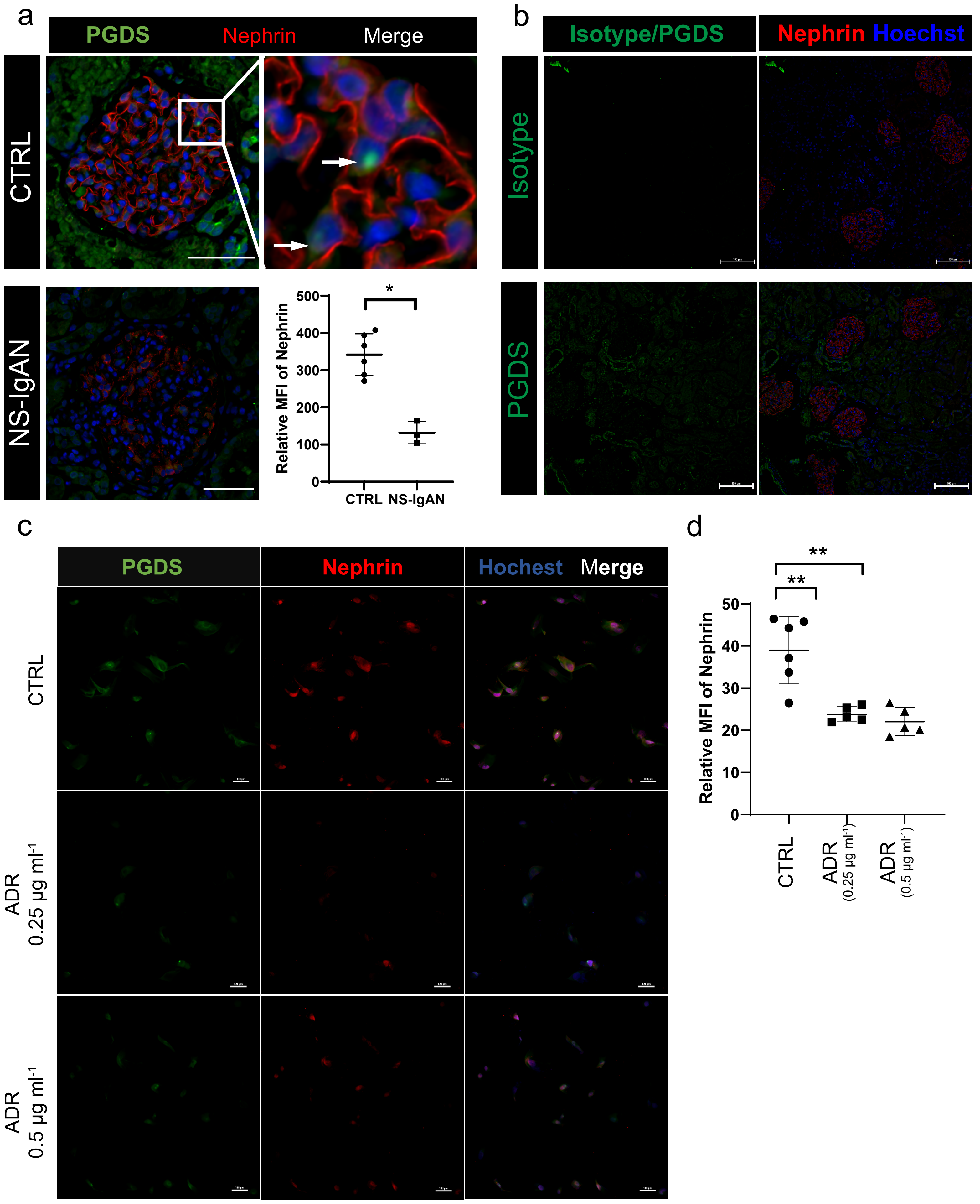

Supplementary figure 8: Expression of PGDS in podocytes. (a) Immunofluorescence staining of PGDS in glomeruli of the NS-IgAN patient and healthy control child, and statistical analysis of PGDS glomerular mean fluorescence intensity quantified using NIS-Elements software. (b) Immunofluorescence staining of PGDS and isotype in kidney of the same healthy control child. (c) Representative pictures of PGDS and nephrin staining in podocytes exposed to vehicle, 0.25μg ml^{-1} ADR and 0.5μg ml^{-1} ADR. Scale bars: 100 μm. (d) Statistical analysis of nephrin mean fluorescence intensity in podocytes.

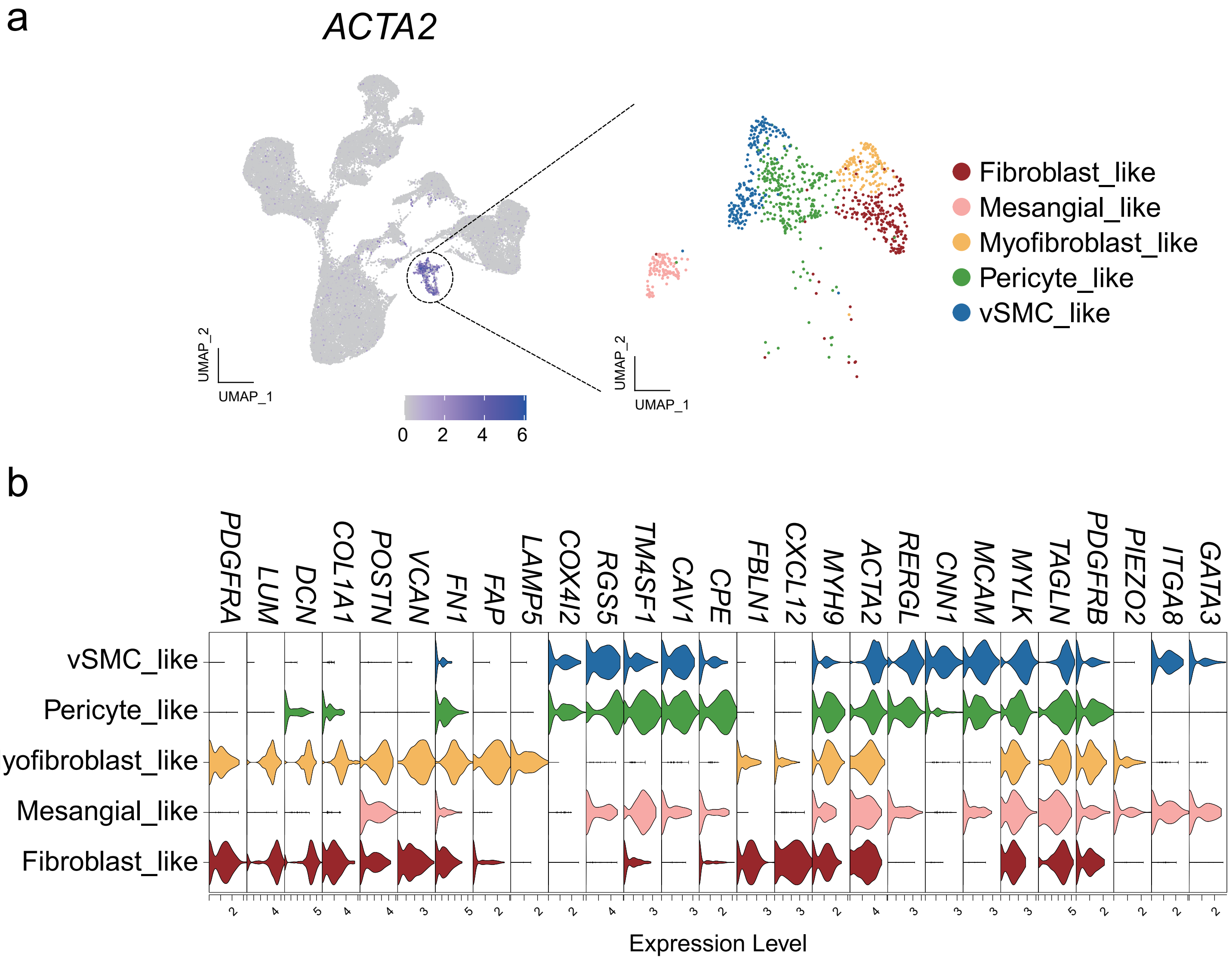

Supplementary figure 9: Identification and characterization of mesenchymal stromal cells (MSCs). (a) Five cell types were visualized by UMAP plotting through reclustering analysis of Cluster 16 in kidney (ACTA2⁺ cells). (b) Violin plots showing marker genes in 5 cell types of MSCs.

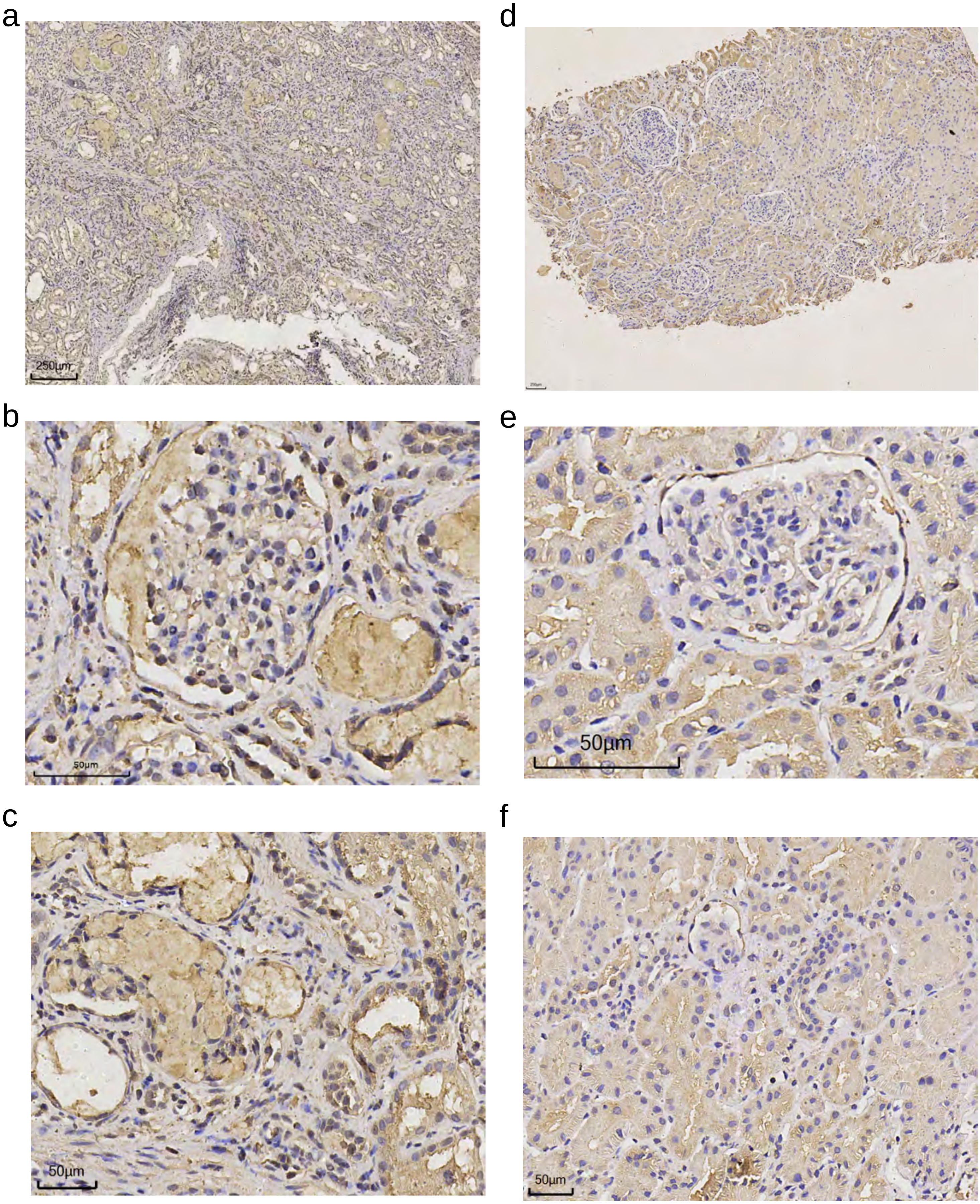

Supplementary figure 10:Expression of PGDS in the kidney from normal children and NS-IgAN patient. (a-c) Immunohistochemistry showing expression of PGDS in kidney (a), glomeruli (b) or tubules (c) in normal children kidney. (d-f) Immunohistochemistry showing expression of PGDS in kidney (d), glomeruli (e) or tubules (f) in patient with NS-IgAN.
